# ﻿Corrections and additions to the catalogue of the bees (Hymenoptera, Anthophila) of Russia

**DOI:** 10.3897/zookeys.1187.113240

**Published:** 2023-12-21

**Authors:** Maxim Yu. Proshchalykin, Alexander V. Fateryga, Yulia V. Astafurova

**Affiliations:** 1 Federal Scientific Center of the East Asia Terrestrial Biodiversity, Far East Branch of the Russian Academy of Sciences, Vladivostok 690022, Russia Federal Scientific Centre for East Asian Terrestrial Biodiversity, Far East Branch of the Russian Academy of Sciences Vladivostok Russia; 2 T.I. Vyazemsky Karadag Scientific Station – Nature Reserve of RAS – Branch of A.O. Kovalevsky Institute of Biology of the Southern Seas of RAS, Nauki Str. 24, Kurortnoye, 298188 Feodosiya, Russia Vyazemsky Karadag Scientific Station – Nature Reserve of RAS – Branch of Kovalevsky Institute of Biology of the Southern Seas of RAS Feodosiya Russia; 3 Zoological Institute, Russian Academy of Sciences, Saint Petersburg 199034, Russia Zoological Institute, Russian Academy of Sciences Saint Petersburg Russia

**Keywords:** Biodiversity, conservation, continental checklist, new record, new synonym, pollinators, taxonomy

## Abstract

The present study is an update to the first catalogue of Russian bees published in 2017. For the Russian fauna, five recently described species are reported, as well as 45 species newly recorded since the first catalogue (including one invasive species), nine species overlooked in this previous Russian checklist, and 17 published synonymies. Original records are provided for nine species previously unknown to Russia and, as a taxonomic act, one species, *Anthidiumovasi* Warncke, 1980, **syn. nov.**, is synonymised with *Icteranthidiumfloripetum* (Eversmann, 1852). Additionally, 14 species are excluded from the original catalogue and numerous other taxonomic changes and clarifications are included. The present work revises the total number of genera for Russia to 64 and the total number of species to 1,268.

## ﻿Introduction

The ‘Annotated Catalogue of Russian bees’ (Astafurova and Proshchalykin 2017; Levchenko et al. 2017; Proshchalykin 2017a; Proshchalykin et al. 2017; Proshchalykin and Astafurova 2017; Proshchalykin and Fateryga 2017), is a major milestone in the study of this diverse group of hymenopteran insects in a vast territory such as that of Russia. Due to the intensive work of the team of authors, it was possible to include all the published data on bees from Russia known at that time in the catalogue. In total, the catalogue contained 1,215 species from 66 genera and six families (Colletidae – 100 species/2 genera; Andrenidae – 244/5; Halictidae – 263/13; Melittidae – 25/3; Megachilidae – 198/18; Apidae – 385/25).

For such works, it has become common practice to publish corrections and additions accumulated over time approximately once every five years. Similar updates have already been released twice for the European Bee Checklist (Rasmont et al. 2017; Ghisbain et al. 2023), first published in 2014 (Nieto et al. 2014). This first update of the catalogue of Russian bees allows for the correction of previous errors, the introduction of the latest nomenclatural and taxonomic changes, as well as the inclusion of taxa recorded for the first time and species newly described for science from this territory.

## ﻿Materials and methods

Bringing together new literature records and taxonomic updates for this work was made possible by (i) an exhaustive review of the literature published since the first catalogue of Russian bees (Astafurova and Proshchalykin 2017; Levchenko et al. 2017; Proshchalykin 2017a; Proshchalykin et al. 2017; Proshchalykin and Astafurova 2017; Proshchalykin and Fateryga 2017), (ii) an in-depth revision of the literature not considered in the catalogue, and (iii) original information provided by the authors of the present work. This new list is mostly based on material directly examined by taxonomists and does not include data published online that has not otherwise been validated by experts (e.g., observations reported on iNaturalist, Discover Life, GBIF).

### ﻿How to use the updated list

The species are ordered by family and listed alphabetically within the following sections:

- Species recently described as new to science (i.e., new species described after 2017);
- Published synonymies (i.e., synonymies published after 2017);
- Other taxonomic changes and clarifications (i.e., relevant changes published after 2017, such as new combinations, taxa upgraded to species rank or downgraded to subspecies rank, as well as clarifications of interesting cases that have led to changes in the updated checklist of the Russian bees);
- Species recorded in Russia after 2017 (i.e., published as new to Russia but not new to science);
- Species overlooked in the Russian catalogue (i.e., species recorded in Russia before 2017 but not included in the annotated catalogue of 2017);
- New species records for Russia (new entries presented in this article for the first time);
- Species to be excluded from the Russian checklist (discussions and explanations of the exclusion of certain species from this new checklist).

The systematics at family, subfamily and tribe levels are mainly based on Michener (2007) and Ascher and Pickering (2023). Generic and subgeneric classifications are generally consistent with those used by Ghisbain et al. (2023), except in some cases noted in the text. In the dating of Morawitz’s species, we follow Kerzhner (1984) and Ebmer (2021). The names “Radoszkowski”, “Lepeletier de Saint-Fargeau”, and “Audinet-Serville” are standardised here, since these authors’ names were originally written variably in different articles. The acronyms for institutions that loaned specimens or provided photographs used in this study are as follows:

**CAFK** research collection of Alexander V. Fateryga, Feodosiya, Russia.

**CMKH** research collection of Max Kasparek, Heidelberg, Germany.

**ETHZ**Entomological Collection of ETH Zurich, Switzerland.

**FSCV** Federal Scientific Center of the East Asia Terrestrial Biodiversity, Far Eastern Branch of the Russian Academy of Sciences, Vladivostok, Russia.

**ISZP**Institute of Systematics and Evolution of Animals, Polish Academy of Sciences, Krakow, Poland.

**OLBL**Oberösterreichisches Landesmuseum, Biologiezentrum, Linz, Austria.

**ZISP**Zoological Institute of the Russian Academy of Sciences, Saint Petersburg, Russia.

**ZMMU**Zoological Museum of the Moscow State University, Russia.

## ﻿Taxonomic updates of the wild bee fauna of Russia

### ﻿Family Colletidae Lepeletier de Saint-Fargeau, 1841

Species recently described as new to science

***Colletesravuloides* Kuhlmann & Proshchalykin, 2023**

*Colletesravuloides* Kuhlmann & Proshchalykin in Proshchalykin and Kuhlmann 2023: 37, ♂ (holotype: ♂, Russia, Tuva Republic, 11 km W of Ust’-Elegest, steppe, 27.VII.2018, S. Luzyanin, D. Sidorov, ZISP).

**Distribution.** Russia (Eastern Siberia: Tuva Republic).

#### ﻿Published synonymies

**Hylaeus (Hylaeus) montivagus Dathe, 1986**

**Notes.** Synonymised with *Hylaeustsingtauensis* (Strand, 1915), which is the senior synonym according to Proshchalykin and Dathe (2018: 582).

#### ﻿Species recorded in Russia after 2017

***Colletesasiaticus* Kuhlmann, 1999**

**Distribution.** First recorded for Russia (North Caucasus: Dagestan Republic) by Proshchalykin and Kuhlmann (2019: 162). Outside Russia known from Turkey, Azerbaijan, Iran, and Turkmenistan (Proshchalykin 2017b).

***Colletescariniger* Pérez, 1903**

**Distribution.** First recorded for Russia (south of European part: Astrakhan Province) by Proshchalykin and Kuhlmann (2020: 22). Records from Crimea by Filatov (2006: 110) and Filatov et al. (2006: 258) need to be checked. Outside Russia known from Bulgaria, Greece, Turkey, Azerbaijan, Israel, Jordan, Lebanon, Syria, Libya, and Egypt (Proshchalykin 2017b).

***Colletesconradti* Noskiewicz, 1936**

**Distribution.** First recorded for Russia (south of European part: Astrakhan Province) by Proshchalykin and Kuhlmann (2020: 22). Outside Russia known from Uzbekistan, Kyrgyzstan, Tajikistan, Kazakhstan, and China (Qinghai, Xinjiang) (Proshchalykin 2017b).

***Colletesdorsalis* Morawitz, 1888**

**Distribution.** First recorded for Russia (North Caucasus: Dagestan Republic) by Proshchalykin and Kuhlmann (2019: 162). Outside Russia known from Turkey, Georgia, Armenia, Azerbaijan, Kazakhstan, Uzbekistan, Kyrgyzstan, Turkmenistan, Tajikistan, and Iran (Proshchalykin 2017b).

***Colletesedentulus* Noskiewicz, 1936**

**Distribution.** First recorded for Russia (North Caucasus: Dagestan Republic) by Proshchalykin and Kuhlmann (2019: 161). Outside Russia known from Georgia, Armenia, Azerbaijan, Turkey, Mongolia, and Turkmenistan (Proshchalykin and Kuhlmann 2018).

***Colleteshethiticus* Warncke, 1978**

**Distribution.** First recorded for Russia (North Caucasus: Dagestan Republic) by Proshchalykin and Kuhlmann (2019: 162). Outside Russia known from Romania, Bulgaria, Greece, Turkey, and Azerbaijan (Proshchalykin 2017b).

***Colletesuralensis* Noskiewicz, 1936**

**Distribution.** First recorded for Russia (North Caucasus: Dagestan Republic) by Proshchalykin and Kuhlmann (2019: 162). Records from Tuva Republic (Kuhlmann and Proshchalykin 2011: 8) belongs to *Colleteskaszabi* Kuhlmann, 2002 (see Proshchalykin and Kuhlmann 2015: 326). Outside Russia known from Kazakhstan, Tajikistan, and China (Inner Mongolia) (Proshchalykin 2017b).

***Colleteswollmanni* Noskiewicz, 1936**

**Distribution.** First recorded for Russia (North Caucasus: Dagestan Republic) by Proshchalykin and Kuhlmann (2019: 161). Outside Russia known from Azerbaijan, Kazakhstan, Kyrgyzstan, Uzbekistan, Turkmenistan, Tajikistan, Iran, Pakistan, and China (Proshchalykin and Kuhlmann 2018).

**Hylaeus (Dentigera) breviceps Morawitz, 1876**

**Distribution.** First recorded for Russia (North Caucasus: Dagestan Republic) by Proshchalykin and Dathe (2021: 174). Outside Russia known from the Caucasus, Central Asia, and China (Proshchalykin and Dathe 2021).

**Hylaeus (Dentigera) imparilis Förster, 1871**

**Distribution.** First recorded for Russia (North Caucasus: Dagestan Republic) by Proshchalykin and Dathe (2021: 174). Outside Russia known from the West Palaearctic and Iran (Proshchalykin and Dathe 2021).

**Hylaeus (Dentigera) intermedius Förster, 1871**

**Distribution.** First recorded for Russia (North Caucasus: Dagestan Republic) by Proshchalykin and Dathe (2021: 174). Outside Russia known from the West Palaearctic (Proshchalykin and Dathe 2021).

**Hylaeus (Hylaeus) kotschisus (Warncke, 1981)**

**Distribution.** First recorded for Russia (North Caucasus: Dagestan Republic) by Proshchalykin and Dathe (2021: 176). Outside Russia known from the East Mediterranean, the Caucasus, and Turkey (Proshchalykin and Dathe 2021).

**Hylaeus (Spatulariella) iranicus Dathe, 1980**

**Distribution.** First recorded for Russia (North Caucasus: Dagestan Republic) by Proshchalykin and Dathe (2021: 181). Outside Russia known from the Caucasus, Turkey, and Iran (Proshchalykin and Dathe 2021).

#### ﻿Species overlooked in the previous Russian checklist

***Colletesbrevigena* Noskiewicz, 1936**

**Distribution.** First recorded for Russia (Crimea) by Proshchalykin and Kuhlmann (2012: 25). Outside Russia known from Portugal, Spain, France, Austria, Hungary, Italy, Croatia, North Macedonia, Serbia, Bulgaria, Greece, Cyprus, Turkey, and Azerbaijan (Proshchalykin 2017b).

### ﻿Family Andrenidae Latreille, 1802

#### ﻿Published synonymies

**Andrena (Campylogaster) nova Popov, 1940**

**Notes.** Synonymised with *Andrenachengtehensis* Yasumatsu, 1935, which is the senior synonym according to Astafurova et al. (2023: 418).

**Andrena (Leimelissa) ispida Warncke, 1965**

**Notes.** Following de Dalla Torre (1896: 121), both Warncke (1967: 269) and Gusenleitner and Schwarz (2002: 176) incorrectly considered *Andrenafallax* Eversmann, 1852 to be a junior synonym of A. (Notandrena) chrysosceles Kirby, 1802. However, the lectotype specimen of *Andrenafallax* is conspecific with another species, *A.ispida* Warncke, 1965. According to Article 23.9.1 of the ICZN (1999), the prevailing usage of “*Andrenaispida*” as a valid name must not be maintained since *A.fallax* Eversmann, 1852 was mentioned as a valid name after 1899 by Popov (1950) and *A.ispida* Warncke, 1965 has been mentioned in fewer than 25 publications (Astafurova et al. 2022a: 400).

**Andrena (Melandrena) gallica Schmiedeknecht, 1883**

**Notes.** Synonymised with *Andrenaassimilis* Radoszkowski, 1876, which is the senior synonym according to Wood and Monfared (2022: 60).

**Andrena (Taeniandrena) similis Smith, 1849**

**Notes.** Synonymised with *Andrenarussula* Lepeletier de Saint-Fargeau, 1841, which is the senior synonym according to Praz et al. (2022: 404).

**Andrena (Andrena) bulgariensis Warncke, 1965**

**Notes.** Synonymised with *Andrenainconstans* Morawitz, 1877, which is the senior synonym according to Wood (2023: 58).

#### ﻿Other taxonomic changes and clarifications

##### Subgeneric classification of *Andrena* Fabricius, 1775

In the last few years, new subgenera have been described and new combinations have been proposed. These changes are included in the current updated list.

Andrena (Campylogaster) incisa Eversmann, 1852 = *A.* (incertae sedis) *incisa* Eversmann, 1852

Andrena (Carandrena) semiflava Lebedev, 1932 = A. (Notandrena) semiflava Lebedev, 1932

Andrena (Didonia) stepposa Osytshnjuk, 1977 = A. (Hamandrena) stepposa Osytshnjuk, 1977

Andrena (Larandrena) sericata Imhoff, 1868 = A. (Leucandrena) sericata Imhoff, 1868

Andrena (Larandrena) ventralis Imhoff, 1832 = A. (Leucandrena) ventralis Imhoff, 1832

Andrena (Poliandrena) altaica Lebedev, 1932 = A. (Ulandrena) altaica Lebedev, 1932

Andrena (Poliandrena) florea Fabricius, 1793 = A. (Bryandrena) florea Fabricius, 1793

Andrena (Poliandrena) limbata Eversmann, 1852 = A. (Limbandrena) limbata Eversmann, 1852

Andrena (Poliandrena) ornata Morawitz, 1866 = *A.* (incertae sedis) *ornata* Morawitz, 1866

Andrena (Poliandrena) polita Smith, 1847 = A. (Ulandrena) polita Smith, 1847

Andrena (Poliandrena) tatjanae Osytshnjuk, 1995 = *A.* (incertae sedis) *tatjanae* Osytshnjuk, 1995

Andrena (Proxiandrena) alutacea E. Stoeckhert, 1942 = A. (Micrandrena) alutacea Stöckhert, 1942

Andrena (Proxiandrena) proxima (Kirby, 1802) = A. (Micrandrena) proxima (Kirby, 1802)

Andrena (Ptilandrena) vetula Lepeletier de Saint-Fargeau, 1841 = A. (Simandrena) vetula Lepeletier de Saint-Fargeau, 1841

Andrena (Thysandrena) hypopolia Schmiedeknecht, 1884 = *A.* (incertae sedis) *hypopolia* Schmiedeknecht, 1884

Andrena (Thysandrena) ranunculorum Morawitz, 1877 = *A.* (incertae sedis) *ranunculorum* Morawitz, 1877

Andrena (Zonandrena) chrysopyga Schenck, 1853 = A. (Melandrena) chrysopyga Schenck, 1853

Andrena (Zonandrena) flavipes Panzer, 1799 = A. (Melandrena) flavipes Panzer, 1799

Andrena (Zonandrena) sibirica Morawitz, 1888 = A. (Melandrena) sibirica Morawitz, 1888

**Andrena (Hoplandrena) scotica Perkins, 1916**

**Notes.** This name replaces the use of *Andrenacarantonica* sensu auctorum; *A.carantonica* Pérez, 1902 is treated as a nomen dubium (Wood et al. 2022: 403).

**Distribution.** Europe, Russia (European part, Urals), Armenia, Azerbaijan, Iran (Gusenleitner and Schwarz 2002).

**Andrena (Plastandrena) aulica Morawitz, 1876**

**Notes.** According to Warncke (1967: 179) and Gusenleitner and Schwarz (2002: 130) *A.aulica* Morawitz, 1876 is a junior synonym of *A.bimaculata* (Kirby, 1802). However, Popov (1949), Osytshnjuk et al. (1978) and Astafurova et al. (2021) regarded *A.aulica* as a valid species. Wood and Monfared (2022: 66) regarded *A.aulica* as a subspecies of *A.bimaculata* (Kirby, 1802). The taxonomic status of *A.bimaculata* sensu lato is problematic and requires a revision. Although Astafurova et al. (2021) reported *A.aulica* from the European part of Russia, the distribution of this species is unclear due to ongoing taxonomic confusion with *A.bimaculata*. In the present update, we do not treat *A.aulica* as a full species.

**Andrena (Taeniandrena) eversmanniana Osytshnjuk, 1994**

**Notes.** Recognised as a valid species (not as a synonym of *Andrenamarginata* Fabricius, 1776) according to Astafurova et al. (2022a: 404).

**Distribution.** Russia (Urals: Orenburg Province), Kazakhstan, and Uzbekistan (Astafurova et al. 2022a).

**Andrena (Taeniandrena) afzeliella (Kirby, 1802)**

**Notes.** Recognised as a valid species (not as a synonym of *Andrenaovatula* Schenck, 1853) according to Praz et al. (2022: 383), *Andrenaafzeliella* here replaces *A.ovatula* sensu auctorum from the 2017 checklist.

**Distribution.** Europe, Egypt, Russia, the Caucasus, Turkey, Israel, Syria, Iraq, Iran, Afghanistan, Central Asia (Praz et al. 2022).

**Andrena (Truncandrena) rufomaculata Friese, 1921**

**Notes.** The reports of this species from Crimea (Proshchalykin et al. 2017: 275) actually referred to *Andrenaoptata* Warncke, 1975 (Wood et al. 2020: 30).

**Distribution.** Eastern Europe, the Balkans, and Turkey. *Andrenarufomaculata* is distributed in Turkey, Iran and the Levant (Wood et al. 2020; Wood and Monfared 2022).

#### ﻿Species recorded in Russia after 2017

**Andrena (Brachyandrena) pinguis Ariana, Scheuchl, Tadauchi & Gusenleitner, 2009**

**Distribution.** First recorded for Russia (south of European part: Volgograd Province) by Wood and Monfared (2022: 105). Outside Russia known from Turkey and Iran (Wood and Monfared 2022).

#### ﻿Species overlooked in the previous Russian checklist

**Andrena (Andrena) fulva (Müller, 1766)**

**Distribution.** First recorded for Russia (north-west of European part: Metgethen, now Kosmodem’yanskoe, Kaliningrad Province) by Möschler (1938: 273). Outside Russia known from Europe and eastern Turkey (Gusenleitner and Schwarz 2002; Wood 2023).

**Andrena (Euandrena) meripes Friese, 1922**

**Distribution.** First recorded for Russia (Eastern Siberia: Irkutsk, as *Andrenanigripes* Friese, 1914, nec Provancher, 1895) by Friese (1914: 225). Outside Russia known from eastern Kazakhstan (Friese 1922).

#### ﻿Species to be excluded from the Russian checklist

***Andrena* (incertae sedis) *lateralis* Morawitz, 1876**

**Distribution.** It was reported from Russia by Astafurova et al. (2022b: 136) on the base of an erroneous record. The species occurs in Europe, the Caucasus, Turkey, Israel, Iran, Afghanistan, Central Asia (Astafurova et al. 2022b).

**Andrena (Truncandrena) albopicta Radoszkowski, 1874**

**Distribution.** It was reported from Russia by Lykov (2008: 32) and Rasmont et al. (2017: 19) on the base of an erroneous record. The species occurs in Armenia, Azerbaijan, Turkey and Iran (Morawitz 1877; Wood and Monfared 2022).

### ﻿Family Halictidae Thomson, 1869

#### ﻿Published synonymies

**Lasioglossum (Hemihalictus) sabulosum (Warncke, 1986)**

**Notes.** Synonymised with *Lasioglossummonstrificum* (Morawitz, 1891), which is the senior synonym according to Pauly and Belval (2017: 27).

***Sphecodesorientalis* Astafurova & Proshchalykin, 2014**

**Notes.** Synonymised with *Sphecodespieli* Cockerell, 1931, which is the senior synonym according to Astafurova et al. (2018: 38).

#### ﻿Other taxonomic changes and clarifications

##### Generic and subgeneric classification of Halictini

The generic and subgeneric classification of Halictini has remained unclear and inconsistent depending on the author or authors. The subgeneric classification of *Halictus* follows Michener (2007). The genus *Seladonia* is not used here, and species included in *Seladonia* in Ghisbain et al. (2023) are placed here in the subgenera *Pachyceble* Moure, 1940, *Seladonia* Robertson, 1918, and *Vestitohalictus* Blüthgen, 1961. The subgeneric classification of *Lasioglossum* is based on the conclusions of Gibbs et al. (2013) and follows Ghisbain et al. (2023) and Ascher and Pickering (2023). Species included in the subgenus Evylaeus in the first catalogue of Russian bees (Astafurova and Proshchalykin 2017) are now split into the subgenera *Biennilaeus* Pesenko, 2007, *Dialictus* Robertson, 1902, *Hemihalictus* Cockerell, 1897, *Pyghalictus* Warncke, 1975, and *Sphecodogastra* Ashmead, 1899.

***Nomiapismonstrosa* (Costa, 1861)**

**Notes.***Nomiapisarmata* (Olivier, 1812) was synonymised with *N.monstrosa* by Baker (2002: 36). We now follow the position that *N.armata* (Olivier, 1812) is a nomen dubium (since was described from the deserts of Arabia, from which *N.monstrosa* has never been recorded).

#### ﻿Species recorded in Russia after 2017

**Lasioglossum (Hemihalictus) medinai (Vachal, 1895)**

**Distribution.** First record for Russia (south of European part: Volgograd Province) by Pauly et al. (2019: 32). Outside Russia known from North Africa, Southern Europe, and Israel (Pauly et al. 2019).

**Lasioglossum (Hemihalictus) adabaschum (Blüthgen, 1931)**

**Distribution.** First record for Russia (south of European part: Astrakhan Province, Kalmykia Republic) by Astafurova and Proshchalykin (2023a: 2). Outside Russia known from Turkmenistan (Astafurova and Proshchalykin 2023a).

#### ﻿New species records for Russia

***Pseudapisbytinski* (Warncke, 1976)**

**Distribution. New record.** Russia, North Caucasus: 2 ♂♂, Dagestan Republic, Kamyshchay River valley, 41°54′29″N, 48°13′59″E, 29.VI.2018, Yu. Astafurova (ZISP). Outside Russia known from Egypt, Israel, Turkey, Armenia, and Azerbaijan (Astafurova 2014).

***Sphecodeskozlovi* Astafurova & Proshchalykin, 2015**

**Distribution. New record** Russia, Far East: 4 ♀♀, Amurskaya Province, Tukuringra Ridge, Zeya Mts., 12.VI.1912, Kozhanchikov (ZMMU); 1 ♂, Primorskiy Territory, Lazo Nature Reserve, 23 km SE of Lazo, 4.IX.1981, Yu. Pesenko (ZISP); 1 ♂, Primorskiy Territory, Suputinka River, 4.VIII.1948, Gussakovskij (ZMMU). Outside Russia known from China (Inner Mongolia, Shanxi, Ningxia) and Mongolia (Dornod, Khentii) (Astafurova et al. 2018).

#### ﻿Species overlooked in the previous Russian checklist

**Lasioglossum (Leuchalictus) majus (Nylander, 1852)**

**Distribution**. Russia, centre of European part: 2 ♀♀, Kursk Province, near Kursk, 4.VI.1916, S. Malyshev (ZISP); 2 ♀♀, Kursk Province, Borisovka, 4.VI.1916, S. Malyshev (ZISP). Pesenko (1986: 113) recorded this species from “south of the European part of the USSR” without giving a precise locality for Russia. The record from Russia (Stavropol Territory) by Chenikalova (2005: 26) needs to be checked. Outside of Russia known from north-western Africa (Tunisia, Algeria), Europe (nearly throughout from Spain in the west as far as northern Germany, Poland), and through Turkey to northern Iran (Ebmer 1988; Pesenko et al. 2000).

### ﻿Family Melittidae Schenck, 1860

#### ﻿Species overlooked in the previous Russian checklist

***Macropisfrivaldszkyi* Mocsáry, 1878**

**Distribution.** First recorded for Russia (Crimea and Eastern Siberia: Krasnoyarsk Territory) by Popov (1958: 502). Outside Russia known from Balkans to Turkey, Syria, and Kazakhstan (Popov 1958; Michez and Patiny 2005).

### ﻿Family Megachilidae Latreille, 1802

#### ﻿Species recently described as new to science

**Hoplitis (Hoplitis) astragali Fateryga, Müller & Proshchalykin, 2023**

*Hoplitisastragali*Fateryga et al. 2023: 664, ♀, ♂ (holotype: ♂, Russia, Dagestan, Levashi district, Tsudakhar, 10.VI.2019, A. Fateryga, ZISP).

**Distribution.** Russia (North Caucasus: Dagestan Republic), Azerbaijan (Nakhchivan Autonomous Republic), and southernmost Turkmenistan.

**Hoplitis (Hoplitis) dagestanica Fateryga, Müller & Proshchalykin, 2023**

*Hoplitisdagestanica*Fateryga et al. 2023: 647, ♀, ♂ (holotype: ♂, Russia, Dagestan, Levashi district, Tsudakhar, 11.VI.2019, A. Fateryga, ZISP).

**Distribution.** Russia (North Caucasus: Dagestan Republic).

#### ﻿Published synonymies

**Coelioxys (Allocoelioxys) conspersus Morawitz, 1873**

**Notes.** Synonymised with *Coelioxyspolycentris* Förster, 1853, which is the senior synonym according to Schwarz and Gusenleitner (2003: 1224). This synonymy was previously overlooked by Proshchalykin and Fateryga (2017) (see also Fateryga et al. 2019).

**Pseudoanthidium (Pseudoanthidium) eversmanni (Radoszkowski, 1886)**

**Notes.** Synonymised with *Pseudoanthidiumtenellum* (Mocsáry, 1880), which is the senior synonym according to Litman et al. (2021: 1313).

**Pseudoanthidium (Pseudoanthidium) reptans (Eversmann, 1852)**

**Notes.** Synonymised with *Pseudoanthidiumnanum* (Mocsáry, 1880), which is the subjective synonym according to Litman et al. (2021: 1296). *Pseudoanthidiumreptans* is a nomen oblitum, while *P.nanum* is a nomen protectum.

#### ﻿Other taxonomic changes and clarifications

##### Subgeneric classification of *Coelioxys* Latreille, 1809

A comprehensive morphological revision of the *Coelioxys* subgenera by da Rocha Filho and Packer (2016) was not followed by Proshchalykin and Fateryga (2017). According to this revision, *Coelioxysalatus* Förster, 1853, *C.elongatus* Lepeletier de Saint-Fargeau, 1841, *C.inermis* (Kirby, 1802), and *C.mandibularis* Nylander, 1848 should be placed in the subgenus Paracoelioxys Gribodo, 1884, *C.aurolimbatus* Förster, 1853 and *C.rufescens* Lepeletier de Saint-Fargeau & Audinet-Serville, 1825 should be placed in the subgenus Rozeniana da Rocha Filho, 2016, and *C.conoideus* (Illiger, 1806) should be placed in the monotypic subgenus Melissoctonia da Rocha Filho, 2016. The subgeneric placement of four species from the Russian fauna was not mentioned by da Rocha Filho and Packer (2016). Based on the material examined from the Primorskiy Territory of Russia, we hereby place *C.pielianus* Friese, 1935 in the subgenus Paracoelioxys and *C.ruficinctus* Cockerell, 1931 in the subgenus Rozeniana. At the same time, the subgeneric placement of *C.lanceolatus* Nylander, 1852 and *C.obtusispina* Thomson, 1872 still remains uncertain (see also Ghisbain et al. 2023).

***Icteranthidiumfloripetum* (Eversmann, 1852)**

Fig. 1A–F

*Anthidiumfloripetum* Eversmann, 1852: 83, ♀, ♂ (lectotype: ♀, “Spask Aug” [Russia: Orenburg Province, Spasskoye], IZSP, designated by Litman et al. (2021: 1300)).

*Anthidiumovasi* Warncke, 1980: 176, ♀, ♂ (holotype: ♀, “Yesilhisar/Kayseri, Türkei” [Turkey], 3.VIII.1979, K. Warncke, OLBL), syn. nov.

**Notes.***Anthidiumfloripetum* was first placed in the genus *Icteranthidium* Michener, 1948 by Litman et al. (2021: 1300). Previously it was treated in the genus *Pseudoanthidium* Friese, 1898 (Proshchalykin and Fateryga 2017: 302) due to an incorrect synonymisation with *P.lituratum* (Panzer, 1801) by Warncke (1980: 161). Kasparek (2022: 168) first published high-quality illustrations of the female holotype of *Icteranthidiumovasi*, which allowed us to ascertain that it is surprisingly almost identical in morphology to the female lectotype of *I.floripetum* (Fig. 1A, C, F). Therefore, these species should be treated as conspecific with Eversmann’s name taking priority. It is also of note that the male paralectotype of *I.floripetum* has the same large reddish-brown maculation in upper gena behind the eye (Fig. 1B, D) as the female types of both *I.floripetum* and *I.ovasi*, while the male paratypes of *I.ovasi* do not have them, according to Kasparek (2022: 168).

**Figure 1.**
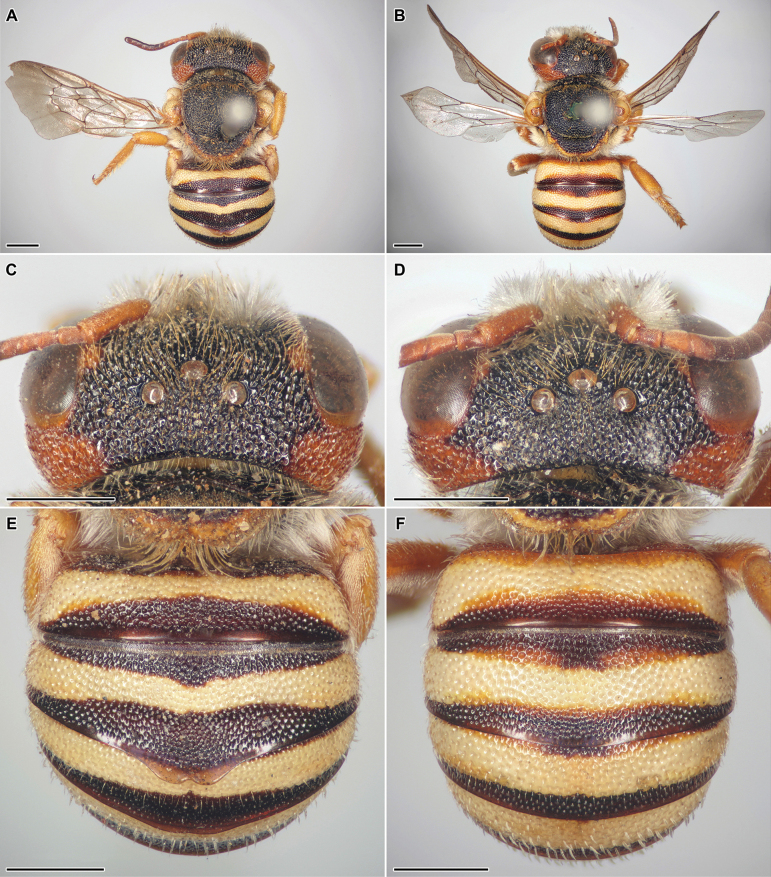
*Icteranthidiumfloripetum* (Eversmann, 1852) **A, C, E** lectotype, female **B, D, F** paralectotype, male **A, B** habitus in dorsal view **C, D** head in dorsal view **E, F** metasoma in dorsal view. Scale bars: 1 mm.

**Distribution.** Russia (Urals: Orenburg Province), Turkey, Iran, and Kazakhstan (Atyrau Province) (Litman et al. 2021; Kasparek 2022).

**Megachile (Chalicodoma) albocristata Smith, 1853**

Fig. 2A

**Notes.** This name replaces the use of *Megachilelefebvrei* sensu Proshchalykin and Fateryga (2017: 305) and references therein. In the narrow sense, *M.lefebvrei* (Lepeletier de Saint-Fargeau, 1841) is present in North Africa and the Iberian Peninsula, and possibly in southern France (Ghisbain et al. 2023). Specimens from Russia were re-identified as *M.albocristata* by Fateryga and Proshchalykin (2020: 228). These species differ in the colour of the vestiture and the nature of the tergal fasciae in the female sex: in *M.lefebvrei*, the vestiture is predominantly grey-white and the tergal fasciae are interrupted medially; in *M.albocristata*, the vestiture is predominantly black, sometimes with spots of white hairs laterally on the terga (Fateryga and Proshchalykin 2020: 228; Ghisbain et al. 2023: 63). The typical form of *M.albocristata* occurs in Crimea, while a form from Dagestan has some traits intermediate with *M.hungarica* Mocsáry, 1877 (Fateryga and Proshchalykin 2020: 228). The taxonomy of this species complex, known as the *lefebvrei* group (*M.lefebvrei*, *M.hungarica*, *M.albocristata*, as well as *M.lucidifrons* Ferton, 1905 and *M.roeweri* (Alfken, 1927)), requires further investigation (Ghisbain et al. 2023: 63).

**Figure 2.**
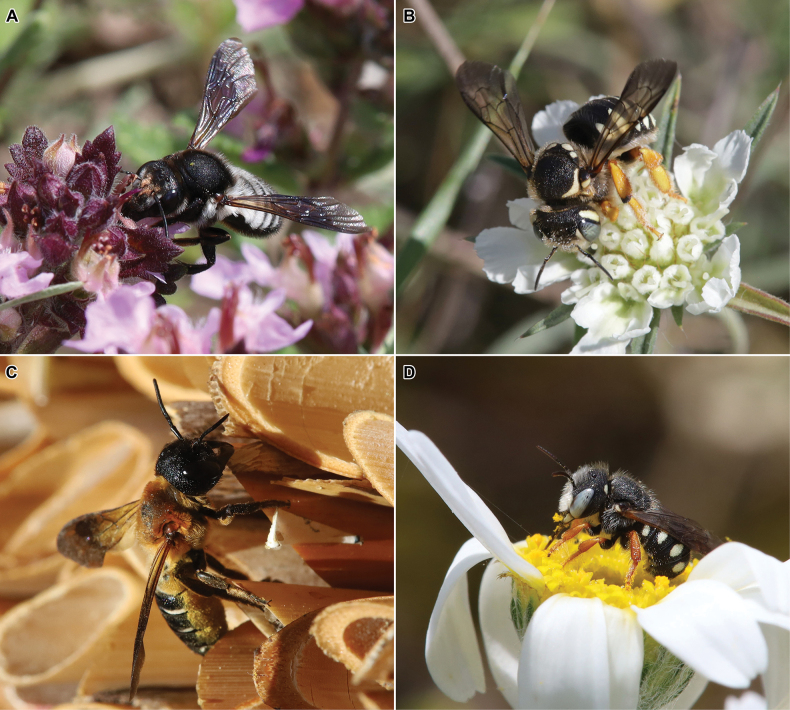
Some species of bees recently reported from Russia **A** female of *Megachilealbocristata* Smith, 1853 at flower of *Teucriumchamaedrys* L. (Lamiaceae), Dagestan Republic, 13.VI.2021 **B** female of *Trachusaintegra* (Eversmann, 1852) on inflorescence of *Lomelosiaargentea* (L.) Greuter & Burdet (Caprifoliaceae), Crimea, 10.VII.2023 **C** female of *Megachilesculpturalis* Smith, 1853 at her nest, Crimea, 24.VII.2021 **D** male of *Pseudoanthidiumstigmaticorne* (Dours, 1873) on inflorescence of *Anthemisruthenica* M. Bieb. (Asteraceae), Crimea, 5.VI.2021. Photographs by A. Fateryga.

**Distribution.** Russia (North Caucasus, Crimea), south-eastern Europe, Georgia, Azerbaijan, Turkey, and Iran (Fateryga and Proshchalykin 2020; Maharramov et al. 2021; Ghisbain et al. 2023).

**Megachile (Eutricharaea) argentata (Fabricius, 1793)**

**Notes.** This species was confirmed as the senior synonym of the widespread species *Megachilepilidens* Alfken, 1924 (Praz and Bénon 2023: 167; Ghisbain et al. 2023: 64).

**Distribution.** Russia (European part, Urals, Western Siberia), Western, Southern, and Eastern Europe, North Africa, Georgia, Armenia, Azerbaijan, Turkey, Jordan, Israel, Iran, and Kazakhstan (Maharramov et al. 2021; Praz and Bénon 2023).

**Trachusa (Paraanthidium) integra (Eversmann, 1852)**

Fig. 2B

**Notes.** Recognised as a valid species (not as a synonym of *Trachusainterrupta* (Fabricius, 1781)) according to Kasparek (2020: 22). In the narrow sense, *T.interrupta* is a mainly Mediterranean species distributed from southern Spain and France, southern Switzerland and Austria over the Balkans to Greece and western Turkey; in south-eastern and Eastern European countries, the distribution extends to Slovakia, Hungary, Romania, and Ukraine (Kasparek 2020, 2022).

**Distribution.** Russia (south of European part, North Caucasus, Crimea), France, Albania, North Macedonia, Greece, Bulgaria, and Turkey (Kasparek 2020, 2022).

#### ﻿Species recorded in Russia after 2017

**Anthidium (Anthidium) melanopygum Friese, 1917**

**Distribution.** First recorded for Russia (North Caucasus: Dagestan Republic, as *Anthidiumspiniventremelanopygum*) by Fateryga et al. (2019: 1167). *Anthidiummelanopygum* is currently treated as a distinct species, not a subspecies of *A.spiniventre* Friese, 1899 (Kasparek and Fateryga 2023: 567). Outside Russia known from Greece, Bulgaria, Turkey, Armenia, Azerbaijan, Lebanon, Iran, and Turkmenistan (Kasparek 2022; Kasparek and Fateryga 2023).

**Coelioxys (Allocoelioxys) acanthura (Illiger, 1806)**

**Distribution.** First recorded for Russia (North Caucasus: Dagestan Republic) by Fateryga et al. (2019: 1169). Outside Russia known from Europe, North Africa, Georgia, Turkey, Cyprus, Israel, Iran, Turkmenistan, Uzbekistan, Kyrgyzstan, Kazakhstan, and China (Fateryga et al. 2019; Ascher and Pickering 2023).

**Coelioxys (Allocoelioxys) mielbergi Morawitz, 1880**

**Distribution.** First recorded for Russia (south of European part: Volgograd Province) by Fateryga and Proshchalykin (2020: 228). Outside Russia known from Uzbekistan, Turkmenistan, and Tajikistan (Fateryga and Proshchalykin 2020).

**Coelioxys (Liothyrapis) decipiens (Spinola, 1838)**

**Distribution.** First recorded for Russia (North Caucasus: Dagestan Republic) by Fateryga et al. (2019: 1171). Outside Russia known from North Africa, Greece, Turkey, Israel, Yemen, Oman, Iran, Iraq, Turkmenistan, Tajikistan, Uzbekistan, Kyrgyzstan, Kazakhstan, China, India, Myanmar, and Thailand (Fateryga et al. 2019; Ascher and Pickering 2023).

**Hoplitis (Alcidamea) beijingensis Wu, 1987**

**Distribution.** First recorded for Russia (Eastern Siberia: Buryatia Republic) by Proshchalykin and Müller (2019: 165). Outside Russia known from northern China (Proshchalykin and Müller 2019; Müller 2023).

**Hoplitis (Alcidamea) curvipes (Morawitz, 1871)**

**Distribution.** First recorded for Russia (North Caucasus: Dagestan Republic) by Fateryga and Proshchalykin (2020: 226). Outside Russia known from Spain, France, Italy, Greece, Bulgaria, Turkey, Azerbaijan, and Syria (Fateryga and Proshchalykin 2020; Ivanov et al. 2023; Müller 2023).

**Hoplitis (Alcidamea) mollis Tkalců, 2000**

**Distribution.** First recorded for Russia (Crimea) by Fateryga and Ivanov (in press). Outside Russia known from Bulgaria, Azerbaijan, Turkey, Syria, Jordan, Uzbekistan, Kyrgyzstan, and Kazakhstan (Müller 2023).

**Hoplitis (Hoplitis) carinata (Stanek, 1969)**

**Distribution.** First recorded for Russia (Crimea) by Fateryga et al. (2019: 1168). Outside Russia known from Greece, Croatia, North Macedonia, Bulgaria, Armenia, Azerbaijan, Turkey, Syria, Jordan, and Iran (Fateryga et al. 2019; Müller 2023).

**Hoplitis (Hoplitis) kaszabi Tkalců, 2000**

**Distribution.** First recorded for Russia (Siberia: Altai and Buryatia republics) by Proshchalykin and Müller (2019: 168). Outside Russia known from Tajikistan, Kazakhstan, Mongolia, and North China (Proshchalykin and Müller 2019; Müller 2023).

**Hoplitis (Platosmia) inconspicua Tkalců, 1995**

**Distribution.** First recorded for Russia (Siberia: Altai, Khakassia and Tuva republics) by Proshchalykin and Müller (2019: 169). Outside Russia known from Mongolia (Proshchalykin and Müller 2019; Müller 2023).

***Icteranthidiumferrugineum* (Fabricius, 1787)**

**Distribution.** First recorded for Russia (South of European part and North Caucasus: Astrakhan Province, Kalmykia and Dagestan republics) by Fateryga et al. (2019: 1167). Outside Russia known from Southern Europe, West and North Africa, Turkey, Cyprus, Syria, Israel, Saudi Arabia, Yemen, Oman, UAE, Afghanistan, Pakistan, Turkmenistan, Kazakhstan, and China (Fateryga et al. 2019; Ascher and Pickering 2023).

***Lithurgustibialis* Morawitz, 1875**

**Distribution.** First recorded for Russia (North Caucasus: Dagestan Republic) by Fateryga et al. (2019: 1166). Outside Russia known from Southern Europe, North Africa, Azerbaijan, Turkey, Cyprus, Syria, Jordan, Israel, United Arab Emirates, Iraq, Iran, Afghanistan, Pakistan, Turkmenistan, Tajikistan, Uzbekistan, and India (Fateryga et al. 2019; Maharramov et al. 2023).

**Megachile (Callomegachile) sculpturalis Smith, 1853**

Fig. 2C

**Distribution.** First recorded for Russia (Crimea) by Ivanov and Fateryga (2019: 10). Outside Russia known from China (including Taiwan), Korean Peninsula, and Japan; introduced into USA, Canada, Switzerland, Lichtenstein, Germany, Austria, Spain, France, Italy, Slovenia, Serbia, Croatia, Bosnia and Herzegovina, Hungary, Ukraine, and India (Ivanov and Fateryga 2019; Sardar et al. 2021; Lanner et al. 2022; Mulenko et al. 2022).

**Megachile (Chalicodoma) albonotata Radoszkowski, 1886**

**Distribution.** First recorded for Russia (North Caucasus: Dagestan Republic) by Fateryga et al. (2019: 1171). Outside Russia known from Southern Europe, Armenia, Azerbaijan Turkey, Israel, Iran, and Turkmenistan (Fateryga et al. 2019; Maharramov et al. 2021).

**Megachile (Chalicodoma) alborufa Friese, 1911**

**Distribution.** First recorded for Russia (North Caucasus: Karachay-Cherkessia Republic, as *Megachilepyrenaica* (Lepeletier de Saint-Fargeau, 1841)) by Fateryga et al. (2019: 1171), but that report referred to *M.alborufa* (Fateryga and Proshchalykin 2020: 229); also reported as *M.alborufa* from Adygea and North Ossetia – Alania republics by Fateryga and Proshchalykin (2020: 229). These two species are closely related and differ in the colour of the legs, as well as the vestiture and the nature of the tergal fasciae in the female sex: *Megachilealborufa* has reddish legs from tibiae onwards and pale pubescence on terga 1 and 2; in *M.pyrenaica*, the legs are mostly black except reddish tarsi while pale pubescence is developed on terga 1–5. As there are no differences in structural morphology, *M.alborufa* may actually represent just a colour form or a subspecies of *M.pyrenaica* (Fateryga and Proshchalykin 2020). The taxonomy of this species complex requires further investigation. *Megachilealborufa* is known outside Russia from Georgia, Azerbaijan, and Turkey (Fateryga and Proshchalykin 2020). In the narrow sense, *M.pyrenaica* is known from Western and Southern Europe, North Africa, Armenia, Azerbaijan, Turkey, Israel, Tajikistan, and Kazakhstan (Maharramov et al. 2021).

**Megachile (Eutricharaea) burdigalensis Benoist, 1940**

**Distribution.** First recorded for Russia (North Caucasus: Dagestan Republic) by Fateryga et al. (2019: 1171). Outside Russia known from Western and Southern Europe, Georgia, Armenia, Azerbaijan, and Kazakhstan (Fateryga et al. 2019; Maharramov et al. 2021).

**Megachile (Pseudomegachile) flavipes Spinola, 1838**

**Distribution.** First recorded for Russia (North Caucasus: Dagestan Republic) by Fateryga et al. (2019: 1171). Outside Russia known from Greece, North Africa, Armenia, Azerbaijan, Turkey, Cyprus, Syria, Israel, Saudi Arabia, Oman, Iran, Iraq, Afghanistan, Pakistan, Turkmenistan, Tajikistan, Uzbekistan, Kyrgyzstan, and India (Fateryga et al. 2019; Maharramov et al. 2021).

**Megachile (Pseudomegachile) tecta Radoszkowski, 1888**

**Distribution.** First recorded for Russia (south of European part and North Caucasus: Kalmykia and Dagestan republics) by Fateryga et al. (2019: 1173); also known from Western Siberia: Altai Territory from where it was earlier incorrectly reported as *Megachilefarinosa* Smith, 1853 by Byvaltsev et al. (2018) (see below). Outside Russia known from Azerbaijan, Iran, Afghanistan, Turkmenistan, Uzbekistan, Tajikistan, Kyrgyzstan, Kazakhstan, and China (Fateryga et al. 2019; Maharramov et al. 2021).

**Osmia (Helicosmia) cinerea Warncke, 1988**

**Distribution.** First recorded for Russia (North Caucasus: Dagestan Republic) by Fateryga and Proshchalykin (2020: 227). Outside Russia known from Azerbaijan, Turkey, Turkmenistan, and Kyrgyzstan (Fateryga and Proshchalykin 2020; Müller 2023).

**Osmia (Hoplosmia) ligurica Morawitz, 1868**

**Distribution.** First recorded for Russia (North Caucasus: Dagestan Republic) by Fateryga and Proshchalykin (2020: 227). Outside Russia known from Western, Southern, and Eastern Europe, North Africa, Georgia, Armenia, Azerbaijan, Turkey, Cyprus, Syria, Jordan, Israel, Iran, and Turkmenistan (Fateryga and Proshchalykin 2020; Müller 2023).

**Osmia (Pyrosmia) cyanoxantha Pérez, 1879**

**Distribution.** First recorded for Russia (North Caucasus: Dagestan Republic) by Fateryga and Proshchalykin (2020: 228); also known from Crimea (Fateryga and Ivanov in press). Outside Russia known from Western, Southern, and Eastern Europe, North Africa, Armenia, Azerbaijan, Turkey, Cyprus, Syria, Jordan, Israel, and Iran (Fateryga and Proshchalykin 2020; Müller 2023).

**Osmia (Pyrosmia) hellados van der Zanden, 1984**

**Distribution.** First recorded for Russia (Crimea) by Fateryga and Ivanov (in press). Outside Russia known from Southern and Eastern Europe, Georgia, Azerbaijan, Turkey, Cyprus, Jordan, and Israel (Müller 2023).

**Protosmia (Protosmia) glutinosa (Giraud, 1871)**

**Distribution.** First recorded for Russia (North Caucasus: Dagestan Republic) by Fateryga and Proshchalykin (2020: 228). Outside Russia known from Western, Southern, and Eastern Europe, North Africa, Azerbaijan, Turkey, Cyprus, Syria, Jordan, Lebanon, Israel, and Iran (Fateryga and Proshchalykin 2020; Müller 2023).

**Pseudoanthidium (Pseudoanthidium) stigmaticorne (Dours, 1873)**

Fig. 2D

**Distribution.** First recorded for Russia (Crimea and North Caucasus: Dagestan Republic) by Litman et al. (2021: 1307). It was also reported earlier from Crimea as Pseudoanthidiumsp. aff.nanum (Mocsáry, 1880) by Fateryga et al. (2018: 243). Outside Russia known from Western, Southern, and Eastern Europe, North Africa, Azerbaijan, Turkey, Cyprus, Syria, Jordan, Israel, Iran, and Turkmenistan (Litman et al. 2021).

#### ﻿Species overlooked in the previous Russian checklist

**Coelioxys (Allocoelioxys) argenteus Lepeletier de Saint-Fargeau, 1841**

**Distribution.** First recorded for Russia (North Caucasus: Dagestan Republic, as *Coelioxysconstrictus* Förster, 1853) by Morawitz (1873: 185) but this record was overlooked by Proshchalykin and Fateryga (2017) (see also Fateryga et al. 2019); also reported from the south of European part: Astrakhan Province (Fateryga et al. 2019: 1171). Outside Russia known from Western, Southern, and Eastern Europe, North Africa, the Caucasus, Turkey, Cyprus, Syria, Jordan, Israel, Iran, Turkmenistan, Tajikistan, Uzbekistan, Kyrgyzstan, Kazakhstan, and China (Fateryga et al. 2019; Ascher and Pickering 2023).

**Megachile (Megachile) pyrenaea Pérez, 1890**

**Distribution.** First recorded for Russia (north-west and north of European part: Leningrad Province and Karelia Republic) by Elfving (1968: 37) but this record was overlooked by Proshchalykin and Fateryga (2017). Outside Russia known from Europe, Armenia, and Turkey (Ascher and Pickering 2023).

**Pseudoanthidium (Exanthidium) eximium (Giraud, 1863)**

**Distribution.** First recorded for Russia (North Caucasus: Ingushetia Republic) by Mavromoustakis (1948: 175) but this record was overlooked by Proshchalykin and Fateryga (2017) (see also Kasparek 2021). Outside Russia known from Portugal in the west across the Mediterranean, Turkey and the Caucasus to the Iranian Elburz Mountains (Kasparek 2021).

#### ﻿New species records for Russia

**Anthidiellum (Anthidiellum) troodicum Mavromoustakis, 1949**

**Distribution. New record** Russia, North Caucasus: 1 ♀, 1 ♂, Dagestan Republic, vicinity of Talgi, 42°52′36″N, 47°26′42″E, on *Teucriumcanum*, 18.VI.2021, A. Fateryga (CAFK). Outside Russia known from Croatia, Greece, Bulgaria, Azerbaijan, Turkey, Cyprus, Syria, Jordan, Lebanon, and Israel (Kasparek 2022; Kasparek et al. 2023).

**Anthidium (Anthidium) dalmaticum Mocsáry, 1884**

**Distribution. New record** Russia, North Caucasus: 6 ♂, Dagestan Republic, vicinity of Talgi, 42°52′36″N, 47°26′42″E, 13.VI.2021, A. Fateryga; 2 ♀, idem, on *Teucriumcanum*, 13.VI.2021, A. Fateryga (1 ♀, 4 ♂ CAFK; 1 ♀, 2 ♂ CMKH). Outside Russia known from the eastern part of the Adriatic Sea (Croatia), Greece, Bulgaria, Turkey, and the Levant to the Caucasus and Iran; also reported from Afghanistan (Kasparek 2022). Specimens from Dagestan resemble the subspecies *A.dalmaticumsyriacum* Pérez, 1912.

**Hoplitis (Alcidamea) ozbeki Tkalců, 2000**

**Distribution. New record** Russia, North Caucasus: 1 ♀, North Ossetia – Alania Republic, Tsey Gorge, 42°47′38″N, 43°54′54″E, on *Leontodon* sp., 30.VI.2021, A. Fateryga (CAFK); 1 ♀, 1 ♂, Dagestan Republic, 3 km NW Khotoch, 42°25′38″N, 46°55′44″E, on *Medicagoglutinosa*, 17.VI.2023, A. Fateryga (CAFK). Outside Russia known from Georgia and Turkey (Müller 2023).

**Hoplitis (Hoplitis) linguaria (Morawitz, 1875)**

**Distribution. New record** Russia, North Caucasus: 1 ♀, Dagestan Republic, Tsudakhar, 42°19′40″N, 47°09′48″E, 11.VI.2019, A. Fateryga (CAFK); 2 ♀, 1 ♂, idem, 16.VI.2021, S. Ivanov (1 ♀ CAFK; 1 ♀, 1 ♂ ETHZ); 1 ♀, idem, on *Onosmacaucasica*, 16.VI.2021, A. Fateryga (CAFK); 1 ♂, idem, 20.VI.2021, A. Fateryga (CAFK); 2 ♀, 1 ♂, idem, 20.VI.2021, S. Ivanov (CAFK); 1 ♀, idem, on *Onosmacaucasica*, 28.VI.2021, A. Fateryga (CAFK); 1 ♂, idem, 29.V.2022, A. Fateryga (CAFK); 5 ♀, idem, on *Onosmacaucasica*, 15.VI.2023, A. Fateryga (CAFK). Outside Russia known from Georgia and Turkey (Müller 2023).

**Megachile (Eutricharaea) anatolica Rebmann, 1968**

**Distribution. New record** Russia, south of European part: 2 ♂, Astrakhan Province, 13 km S Liman, 24–26.VII.2015, M. Proshchalykin, V. Loktionov, M. Mokrousov, S. Belokobylskij (FSCV); 1 ♂, 35 km NNW Astrakhan, 26.VII.2015, M. Proshchalykin, V. Loktionov, M. Mokrousov, S. Belokobylskij (FSCV); 2 ♂, Kalmykia Republic, 17 km SWW Artezian, Kuma River, 18–21.VII.2015, M. Proshchalykin, V. Loktionov, M. Mokrousov, S. Belokobylskij (CAFK; FSCV); 1 ♂, 22 km E Yashkul, 16–18.VII.2015, M. Proshchalykin, V. Loktionov, M. Mokrousov, S. Belokobylskij (FSCV). Outside Russia known from Italy, Greece, Croatia, Turkey, Cyprus, Jordan, Lebanon, Israel, and Iran (Ascher and Pickering 2023; Praz and Bénon 2023).

#### ﻿Species to be excluded from the Russian checklist

**Hoplitis (Alcidamea) laboriosa (Smith, 1878)**

**Distribution.** Was reported on the base of an erroneous record (based on a locality misinterpretation). The species occurs in Kazakhstan, Mongolia, and China (Ghisbain et al. 2023; Müller 2023).

**Hoplitis (Alcidamea) turcestanica (Dalla Torre, 1896)**

**Distribution.** This species was earlier reported from Russia as *Hoplitiscaularis* (Morawitz, 1875) (Proshchalykin and Fateryga 2017; Fateryga et al. 2018), which was considered a senior synonym of *H.turcestanica* (Ungricht et al. 2008). Then, *H.turcestanica* was reinstated as a valid species by Fateryga and Proshchalykin (2020: 226), who provided an additional record from the south of European part: Astrakhan Province. Although, *H.turcestanica* and *H.caularis* are indeed two very different species, the material from Crimea, reported as *H.caularis*, belongs not to *H.turcestanica* but to *H.mollis* (Fateryga and Ivanov in press; see also above), while specimens from the Astrakhan Province belong to an apparently undescribed species (A. Müller, personal communication). *Hoplitisturcestanica* is confirmed to Turkmenistan, Tajikistan, Kyrgyzstan, and Kazakhstan, while *H.caularis* is known from Kazakhstan (Müller 2023). The records of both species from Turkey, Syria, Uzbekistan, and China require confirmation, as are the records of *H.turcestanica* from the North Caucasus and Urals mentioned by Proshchalykin and Fateryga (2017) and Fateryga and Proshchalykin (2020).

**Hoplitis (Anthocopa) taurica (Radoszkowski, 1874)**

**Notes.***Pseudosmiataurica* Radoszkowski, 1874 is considered to be a nomen dubium by Müller (2023) based on the poor description and the unavailability of the type material.

**Hoplitis (Hoplitis) ravouxi (Pérez, 1902)**

**Distribution.** The reports of this species from Crimea (Proshchalykin and Fateryga 2017; Fateryga et al. 2018) actually referred to *Hoplitiscarinata* (Stanek, 1969) (Fateryga et al. 2019) (see above). *Hoplitisravouxi* is distributed in Western, Southern, and Eastern Europe (Müller 2023).

**Hoplitis (Pentadentosmia) nitidula (Morawitz, 1877)**

**Distribution.** Was reported on the base of an apparently erroneous record (based on a locality misinterpretation). The species occurs in Armenia, Iran, Pakistan, Turkmenistan, Uzbekistan, and Kazakhstan (Ghisbain et al. 2023; Müller 2023).

**Osmia (Helicosmia) cyanescens Morawitz, 1875**

**Distribution.** Was reported on the base of an erroneous record (based on a locality misinterpretation). The species occurs in Tajikistan, Kyrgyzstan, and Kazakhstan (Ghisbain et al. 2023; Müller 2023).

**Osmia (Hemiosmia) difficilis Morawitz, 1875**

**Distribution.** Was reported on the base of an erroneous record (based on a locality misinterpretation). The species occurs in Azerbaijan, Turkey, Syria, Lebanon, Israel, Iran, Tajikistan, Uzbekistan, Kyrgyzstan, and Kazakhstan (Müller 2020, 2023).

**Osmia (Osmia) melanocephala Morawitz, 1875**

**Distribution.** Was reported on the base of an erroneous record (based on a locality misinterpretation). The species occurs in Turkmenistan, Tajikistan, Uzbekistan, Kyrgyzstan, Kazakhstan, Mongolia, and China (Müller 2023).

**Osmia (Pyrosmia) gallarum Spinola, 1808**

**Distribution.** The reports of this species from Crimea (Proshchalykin and Fateryga 2017; Fateryga et al. 2018) actually referred to *Osmiahellados* van der Zanden, 1984 (Fateryga and Ivanov in press) (see above). *Osmiagallarum* is distributed in Western, Southern, and Eastern Europe, North Africa, and Turkey (Müller 2023).

**Megachile (Pseudomegachile) farinosa Smith, 1853**

**Distribution.** First recorded for Russia (North Caucasus: Dagestan Republic, as *Megachilederasa* Gerstäcker, 1869) by Morawitz (1873: 149). This record was overlooked by Proshchalykin and Fateryga (2017) (see also Fateryga et al. 2019). An additional report of this species was made by Byvaltsev et al. (2018) from Western Siberia: Altai Territory. All these records, however, referred to *M.tecta* (see above). *Megachilefarinosa* is distributed in East Mediterranean (Greece, Turkey, Cyprus), Israel north of the Dead Sea, Middle East, and Iran (Dorchin and Praz 2018).

### ﻿Family Apidae Latreille, 1802

#### ﻿Species recently described as new to science

***Epeolusasiaticus* Astafurova & Proshchalykin, 2022**

*Epeolusasiaticus*Astafurova and Proshchalykin 2022a: 309, ♀, ♂ (holotype: ♀, Mongolia, Terkhin-Gol, Chulut and Khoit Rivers, 30.VI.1975, E. Narchuk, ZISP). Paratypes from Russia (Altai Republic).

**Distribution.** Russia (Siberia: Altai Republic, Tuva Republic, Zabaikalskiy Territory), Mongolia (Arkhangai, Bayankhongor, Bayan-Ölgii, Dornod, Dornogovi, Govi-Altai, Khuvsgul, Omnogovi, Selenge, Sukhbaatar, Tuv, Ulaanbaatar, Uvs, Uvurkhangai, Zavkhan).

***Epeolusrasmonti* Astafurova & Proshchalykin, 2022**

*Epeolusrasmonti*Astafurova and Proshchalykin 2022b: 202, ♀, ♂ (holotype: ♀, Russia, Buryatia Republic, Gusinoye Lake, Baraty, 25.VII.2007, A. Lelej, M. Proshchalykin, V. Loktionov, ZISP).

**Distribution.** Russia (Eastern Siberia: Buryatia Republic), Mongolia (Bulgan, Dornod, Khentii, Sukhbaatar), China (Beijing).

#### ﻿Published synonymies

**Anthophora (Anthophora) salviae (Panzer, 1805)**

**Notes.** Synonymised with *Anthophoracrinipes* Smith, 1854, which is the valid name according to Maghni et al. (2017: 5). The latter authors considered the basionym *Lasiussalviae* Panzer, 1805 a nomen dubium (Ghisban et al. 2023: 26).

**Anthophora (Paramegilla) prshewalskyi Morawitz, 1880**

**Notes.** Synonymised with *Anthophorasegnis* Eversmann, 1852 (not a synonym of *A.podagra* Lepeletier de Saint-Fargeau, 1841), which is the senior synonym according to Ghisban et al. (2023: 27).

**Eucera (Eucera) eucnemidea Dours, 1873**

**Notes.** Synonymised with *Euceragrisea* Fabricius, 1793, which is the senior synonym according to Dorchin (2023: 12).

**Eucera (Pareucera) nigrita Friese, 1895**

**Notes.** Synonymised with *Euceraalbofasciata* Friese, 1895, which is the senior synonym according to Boustani et al. (2021: 123).

**Eucera (Synhalonia) alternans (Brullé, 1832)**

**Notes.***Eucerarufa* (Lepeletier de Saint-Fargeau, 1841), which is the junior synonym, is retained by Dorchin (2023: 23) as the valid name for this species under the principle of name stability. *Eucerarufa* replaces *E.alternans* from the 2017 checklist, and that *E.alternans* auctorum is referred to in present list by *E.ruficollis*.

***Nomadaobscuriceps* Schwarz & Levchenko, 2017**

**Notes.** Synonymised with *Nomadamitaii* Proshchalykin, 2010, which is the senior synonym according to Proshchalykin et al. (2019: 26).

#### ﻿Other taxonomic changes and clarifications

The following nomenclatural changes were proposed by Dorchin (2023): *Tetralonia* Spinola, 1838 is reestablished as genus, including *Tetraloniella* Ashmead, 1899 (Dorchin et al. 2018); Cubitalia Friese, 1911 is treated as subgenus of Eucera Scopoli, 1770; and Synhalonia Patton, 1879 is retained as subgenus of Eucera as in Michener (2007). Therefore, the following three species previously included in the genus *Cubitalia* and 14 species previously included in the genus *Tetraloniella* (Levchenko et al. 2017) are now transferred to the genus *Eucera* and *Tetralonia* respectively: Eucera (Cubitalia) morio Friese, 1911, E. (C.) parvicornis Mocsáry, 1878, E. (C.) tristis Morawitz, 1876, *Tetraloniaalticincta* (Lepeletier de Saint-Fargeau, 1841), *T.dentata* (Germar, 1839), *T.fulvescens* Giraud, 1863, *T.graja* (Eversmann, 1852), *T.inulae* Tkalců, 1979, *T.julliani* (Pérez, 1879), *T.lyncea* Mocsáry, 1879, *T.mitsukurii* Cockerell, 1911, *T.nana* Morawitz, 1873, *T.pollinosa* (Lepeletier de Saint-Fargeau, 1841), *T.salicariae* (Lepeletier de Saint-Fargeau, 1841), *T.scabiosae* (Mocsáry, 1881), *T.strigata* (Lepeletier de Saint-Fargeau, 1841), and *T.vicina* Morawitz, 1876.

**Anthophora (Pyganthophora) erschowi Fedtschenko, 1875**

**Notes.** The type series was revised in ZISP by P. Rasmont (Ghisbain et al. 2023: 26). The specimens comprising the type series are only females, all belonging to the difficult group of *Anthophoraaestivalis* (Panzer, 1801), in which generally only males can be reliably identified. Therefore, the name *Anthophoraerschowi* was considered to be a species inquirenda and removed from the European (including Russian) checklists.

***Apisceranaussuriensis* Ilyasov, Takahashi, Proshchalykin, Lelej & Kwon, 2019**

**Notes.** Recognised as a separate subspecies according to Ilyasov et al. (2019: 310).

**Distribution.** Russia (Far East: Primorskiy and Khabarovsk territories) (Proshchalykin and Sergeev 2020).

**Eucera (Eucera) pollinosa Smith, 1854**

**Notes.** This species was previously referred to as *Eucerachrysopyga* Pérez, 1879 (Levchenko et al. 2017: 320), as when *Eucera* and *Tetraloniella* were treated as a single genus, *Eucerapollinosa* Smith became a junior homonym of *E.pollinosa* (Lepeletier de Saint-Fargeau, 1841). Now that *Tetralonia* is restored as a genus (which also includes *Tetraloniella*), *E.pollinosa* (Lepeletier de Saint-Fargeau) is moved to *Tetralonia*, and *E.pollinosa* Smith is no longer a junior homonym and becomes the senior synonym of *E.chrysopyga* Pérez. *Eucerapollinosa* Smith was made a nomen protectum by Dorchin (2023).

**Bombus (Bombus) czerskianus Vogt, 1911**

**Notes.** Recognised as a separate species (not as a subspecies of *Bombussporadicus* Nylander, 1848) according to Williams (2021: 271).

**Distribution.** Russia (Eastern Siberia, Far East), North Korea, north-eastern China, and Mongolia (Williams 2021).

**Bombus (Melanobombus) alagesianus Reinig, 1930**

**Notes.** Recognised as a valid species (not as a synonym of *Bombuskeriensis* Morawitz, 1887) according to Williams et al. (2020: 81).

**Distribution.** Russia (North Caucasus), Turkey, Georgia, Armenia, and Iran (Williams et al. 2020).

**Bombus (Melanobombus) incertoides Vogt, 1911**

**Notes.** Recognised as a valid species (not as a synonym of *Bombuskeriensis* s. lat.) according to Williams et al. (2020: 87).

**Distribution.** Russia (Siberia: Tuva and Altai republics) and Mongolia (Williams et al. 2020).

**Bombus (Pyrobombus) koropokkrus Sakagami & Ishikawa, 1972**

**Notes.** Recognised as a valid species (not as a synonym of *Bombushypnorum* (Linnaeus, 1802)) according to Williams et al. (2022: 62).

**Distribution.** Russia (Far East: Sakhalin) and Japan (Hokkaido) (Williams et al. 2022).

**Bombus (Thoracobombus) mocsaryi Kriechbaumer, 1877**

**Notes.** The taxon *mocsaryi* Kriechbaumer, 1877 was re-assessed as a subspecies of *Bombuslaesus*Morawitz (1875) by Brasero et al. (2021) based on genetic and semio-chemical analyses.

#### ﻿Species recorded in Russia after 2017

**Anthophora (Lophanthophora) crysocnemis Morawitz, 1877**

**Distribution.** First recorded for Russia (south of European part: Volgograd Province) by Ghisbain et al. (2023: 27). Outside Russia known from Armenia and Kazakhstan (Ghisbain et al. 2023).

***Epeolusmongolicus* Astafurova & Proshchalykin, 2021**

**Distribution.** First recorded for Russia (Eastern Siberia: Tuva Republic) by Astafurova and Proshchalykin (2022a: 324). Outside Russia known from Kyrgyzstan and Mongolia (Bulgan, Zavkhan) (Astafurova and Proshchalykin 2022a).

**Eucera (Synhalonia) distinguenda (Morawitz, 1875)**

**Distribution.** First recorded for Russia (south of European part: Astrakhan Province) by Levchenko (2019: 20). Outside Russia known from Armenia, Iran, Turkmenistan, and Kazakhstan (Morawitz 1875, 1877, 1894; Popov 1967).

***Nomadaminuscula* Noskiewicz, 1930**

**Distribution.** First recorded for Russia (European part) by Smit (2018: 188). Outside Russia known from Europe, Morocco, Algeria, and Tunisia (Smit 2018).

***Nomadasubcornuta* (Kirby, 1802)**

**Distribution.** First recorded for Russia (European part) by Ghisbain et al. (2023: 45). Outside Russia known from United Kingdom, Belgium, Netherlands, Germany, Czech Republic, Hungary, Estonia, and Finland (Ghisbain et al. 2023).

#### ﻿Species overlooked in the previous Russian checklist

***Epeolusnudiventris* Bischoff, 1930**

**Distribution.** Described from Russia (Siberia: Buryatia Republic) by Bischoff (1930: 14). Outside Russia known from Kazakhstan, Uzbekistan, Kyrgyzstan, Turkmenistan, Tajikistan, and Mongolia (Khovd) (Astafurova and Proshchalykin 2023b).

#### ﻿New species records for Russia

***Epeolusruficornis* Morawitz, 1875**

**Distribution. New record** RUSSIA, south of European part: 2 ♀♀, 2 ♂♂, Kalmykia Republic, 17 km SSW Artezian, Kuma River, 2–3.VII.2016, Yu. Astafurova; 1 ♂, Astrakhan Province, 35 km NNW Astrakhan, 26.VII.2015, M. Proshchalykin; 1 ♂, Astrakhan Province, Sedlistoye, 8.VI.1927, Plotnikov (ZISP). Outside Russia known from Azerbaijan, Kazakhstan, Uzbekistan, Kyrgyzstan, Turkmenistan, Tajikistan, Mongolia, and China (Xinjiang, Gansu) (Astafurova and Proshchalykin 2023b).

***Tetraloniayoshihiroi* (Ikudome, 2022)**

**Distribution. New record** Russia, Far East: 1 ♂, Primorskiy Territory, Kamen-Rybolov, 28.VIII.1980, Romankov (FSCV); 1 ♂, Primorskiy Territory, Novokachalinsk, 4.VIII.2006, Belokobylskij (ZISP); 2 ♀♀, idem, 21.VIII.2009, A. Lelej, M. Proshchalykin, V. Loktionov (FSCV). Outside Russia known from Japan (Honshu, Kyushu, Tanegashima), South Korea, and China (Beijing, Zhejiang, Anhui) (Ikudome 2022).

#### ﻿Species to be excluded from the Russian checklist

**Bombus (Melanobombus) keriensis Morawitz, 1887**

**Distribution.** The Russian records of *Bombuskeriensis* in Levchenko et al. (2017: 329) refer to *B.separandus* Vogt, 1909 (Siberia: Tuva and Altai republics) and *B.alagesianus* Reinig, 1930 (North Caucasus) (Williams et al. 2020).

***Thyreusaberrans* (Morawitz, 1875)**

**Notes.** This taxon has been treated as a nomen dubium according to Ghisbain et al. (2023: 28). Records from the European part of Russia must therefore be considered to be unclear due to this taxonomic uncertainty.

## ﻿Conclusions

Here we have presented an update on the knowledge of the species diversity and taxonomy of the bee fauna of Russia, considering all the advances made after the publication of the catalogue of Russian bees (Astafurova and Proshchalykin 2017; Levchenko et al. 2017; Proshchalykin 2017a; Proshchalykin et al. 2017; Proshchalykin and Astafurova 2017; Proshchalykin and Fateryga 2017) and considering material that was overlooked by that work. An updated total of 1,268 species belonging to 64 genera and six families are now recorded within Russia (Table 1, Suppl. material 1).

**Table 1.** Updated species totals for Russian bees.

Family	Subfamily	Tribe	Genus	Number of species
**Colletidae**	**Colletinae**	**Colletini**	*Colletes*	53
	**Hylaeinae**	**Hylaeini**	*Hylaeus*	61
**Andrenidae**	**Andreninae**	**Andrenini**	*Andrena*	231
	**Panurginae**	**Panurgini**	*Camptopoeum*	2
			*Panurginus*	13
			*Panurgus*	1
		**Melliturgini**	*Melitturga*	3
**Halictidae**	**Rophitinae**	–	*Dufourea*	8
			*Flavodufourea*	1
			*Rhophitoides*	1
			*Rophites*	6
			*Systropha*	2
	**Nomiinae**	–	*Lipotriches*	1
			*Nomiapis*	6
			*Pseudapis*	3
	**Nomioidinae**	–	*Ceylalictus*	1
			*Nomioides*	2
	**Halictinae**	**Halictini**	*Halictus*	48
			*Lasioglossum*	150
			*Sphecodes*	38
**Melittidae**	**Dasypodainae**	**Dasypodaini**	*Dasypoda*	8
	**Melittinae**	–	*Macropis*	5
			*Melitta*	13
**Megachilidae**	**Megachilinae**	**Lithurgini**	*Lithurgus*	3
		**Osmiini**	*Chelostoma*	6
			*Heriades*	3
			*Hoplitis*	33
			*Osmia*	44
			*Protosmia*	3
		**Anthidiini**	*Anthidiellum*	2
			*Anthidium*	13
			*Bathanthidium*	2
			*Eoanthidium*	1
			*Icteranthidium*	5
			*Pseudoanthidium*	7
			*Stelis*	14
			*Trachusa*	3
		**Dioxyini**	*Aglaoapis*	1
			*Dioxys*	1
		**Megachilini**	*Coelioxys*	26
			*Megachile*	53
**Apidae**	**Xylocopinae**	**Xylocopini**	*Xylocopa*	6
		**Ceratinini**	*Ceratina*	14
	**Nomadinae**	**Nomadini**	*Nomada*	117
		**Epeolini**	*Epeolus*	17
			*Triepeolus*	2
		**Ammobatoidini**	*Ammobatoides*	2
		**Biastini**	*Biastes*	4
		**Ammobatini**	*Ammobates*	4
			*Parammobatodes*	1
			*Pasites*	2
	**Apinae**	**Osirini**	*Epeoloides*	1
		**Ancylaini**	*Ancyla*	1
		**Ctenoplectrini**	*Ctenoplectra*	1
		**Eucerini**	*Eucera*	36
			*Tetralonia*	16
		**Anthophorini**	*Amegilla*	9
			*Anthophora*	41
			*Habropoda*	1
		**Melectini**	*Melecta*	11
			*Thyreomelecta*	2
			*Thyreus*	9
		**Bombini**	*Bombus*	92
		**Apini**	*Apis*	2
**Total**:	**14 subfamilies**	**27 tribes**	**64 genera**	**1,268 species**

After the revision of the first checklist, we report five species recently described, 45 species newly recorded since the first catalogue (including one species non-native to Russia), nine species overlooked in the previous Russian checklist, and 17 published synonymies. We provide original records for nine species previously unknown to Russia and, as original taxonomic act, we synonymise one species and exclude 14 species from the previous checklist. Numerous other taxonomic changes and clarifications are also included. The final count of species per family, subfamily, tribe and genus is available in Table 1. An updated list of Russian bees is available as Suppl. material 1.

## ﻿Additional information

### Author ORCIDs

Maxim Yu. Proshchalykin https://orcid.org/0000-0001-7870-8226

Alexander V. Fateryga https://orcid.org/0000-0002-5346-3477

Yulia V. Astafurova https://orcid.org/0000-0003-0557-7792

### Data availability

All of the data that support the findings of this study are available in the main text or Supplementary Information.

## Supplementary materials

Supplementary material 1 Updated checklist of the wild bee fauna of Russia Data type doc Explanation note Сhecklist of six families, 14 subfamilies, 27 tribes, 64 genera and 1,268 species of bees from Russia. https://binary.pensoft.net/file/956075This dataset is made available under the Open Database License (http://opendatacommons.org/licenses/odbl/1.0/). The Open Database License (ODbL) is a license agreement intended to allow users to freely share, modify, and use this Dataset while maintaining this same freedom for others, provided that the original source and author(s) are credited.Maxim Yu. Proshchalykin, Alexander V. Fateryga, Yulia V. Astafurova
