# Supplementary material for: ﻿Corrections and additions to the catalogue of the bees (Hymenoptera, Anthophila) of Russia
Source: Zookeys. 2023 Dec 21;1187:301–39. doi: 10.3897/zookeys.1187.113240 (PMC10755745; doi:10.3897/zookeys.1187.113240)
Supplement: Supplementary material 1 — Updated checklist of the wild bee fauna of Russia [file zookeys-1187-301_article-113240__-s001.doc]

**Updated checklist of the wild bee fauna of Russia**

**Family COLLETIDAE Lepeletier** **de Saint-Fargeau, 1841**

**Subfamily Colletinae Lepeletier** **de Saint-Fargeau, 1841**

**Tribe Colletini Latreille, 1802**

**Genus** *Colletes* **Latreille, 1802**

*Colletes albomaculatus* (Lucas, 1849)

*Colletes alini* Kuhlmann, 2000

*Colletes anceps* Radoszkowski, 1891

*Colletes arsenjevi* Kuhlmann, 2006

*Colletes asiaticus* Kuhlmann, 1999

*Colletes brevigena* Noskiewicz, 1936

*Colletes* *cariniger* Pérez, 1903

*Colletes caspicus* Morawitz, 1873

*Colletes chengtehensis* Yasumatsu, 1935

*Colletes cinerascens* Morawitz, 1893

*Colletes collaris* Dours, 1872

*Colletes* *conradti* Noskiewicz, 1936

*Colletes cunicularius* (Linnaeus, 1761)

*Colletes daviesanus* Smith, 1846

*Colletes dorsalis* Morawitz, 1888

*Colletes ebmeri* Kuhlmann, 2002

*Colletes edentulus* Noskiewicz, 1936

*Colletes eous* Morice, 1904

*Colletes floralis* Eversmann, 1852

*Colletes fodiens* (Fourcroy, 1785)

*Colletes friesei* Cockerell, 1918

*Colletes fulvicornis* Noskiewicz, 1936

*Colletes hakkari* Kuhlmann, 2002

*Colletes hederae* Schmidt & Westrich, 1993

*Colletes hethiticus* Warncke, 1978

*Colletes hylaeiformis* Eversmann, 1852

*Colletes impunctatus* Nylander, 1852

*Colletes inexpectatus* Noskiewicz, 1936

*Colletes jankowskyi* Radoszkowski, 1891

*Colletes kaszabi* Kuhlmann, 2002

*Colletes kozlovi* Friese, 1913

*Colletes laevifrons* Morawitz, 1893

*Colletes maidli* Noskiewicz, 1936

*Colletes marginatus* Smith, 1846

*Colletes mlokossewiczi* Radoszkowski, 1891

*Colletes nasutus* Smith, 1853

*Colletes patellatus* Pérez, 1905

*Colletes perforator* Smith, 1869

*Colletes pseudocinerascens* Noskiewicz, 1936

*Colletes ravuloides* Kuhlmann & Proshchalykin, 2023

*Colletes ravulus* Noskiewicz, 1936

*Colletes roborovskyi* Friese, 1913

*Colletes senilis* (Eversmann, 1852)

*Colletes sidemii* Radoszkowski, 1891

*Colletes similis* Schenck, 1853

*Colletes subnitens* Noskiewicz, 1936

*Colletes succinctus* (Linnaeus, 1758)

*Colletes tuberculatus* Morawitz, 1893

*Colletes ulrikae* Kuhlmann, 2002

*Colletes uralensis* Noskiewicz, 1936

*Colletes wacki* Kuhlmann, 2002

*Colletes warnckei* Kuhlmann, 2002

*Colletes wollmanni* Noskiewicz, 1936

**Subfamily Hylaeinae Viereck, 1916**

**Tribe Hylaeini Viereck, 1916**

Genus Hylaeus Fabricius, 1793

*Hylaeus* (*Abrupta*) *cornutus* Curtis, 1831

*Hylaeus* (*Dentigera*) *breviceps* Morawitz, 1876

*Hylaeus* (*Dentigera*) *brevicornis* Nylander, 1852

*Hylaeus* (*Dentigera*) *gredleri* Förster, 1871

*Hylaeus* (*Dentigera*) *imparilis* Förster, 1871

*Hylaeus* (*Dentigera*) *intermedius* Förster, 1871

*Hylaeus* (*Dentigera*) *pallidicornis* Morawitz, 1876

*Hylaeus* (*Hylaeus*) *aborigensis* Dathe, 1994

*Hylaeus* (*Hylaeus*) *altaicus* Dathe, 1986

*Hylaeus* (*Hylaeus*) *angustatus* (Schenck, 1861)

*Hylaeus* (*Hylaeus*) *annulatus* (Linnaeus, 1758)

*Hylaeus* (*Hylaeus*) *cardioscapus* Сockerell, 1924

*Hylaeus* (*Hylaeus*) *communis* Nylander, 1852

*Hylaeus* (*Hylaeus*) *dolichocephalus* Morawitz, 1876

*Hylaeus* (*Hylaeus*) *dorni* Dathe, 1986

*Hylaeus* (*Hylaeus*) *gracilicornis* (Morawitz, 1867)

*Hylaeus* (*Hylaeus*) *hungaricus* (Alfken, 1905)

*Hylaeus* (*Hylaeus*) *kotschisus* (Warncke, 1981)

*Hylaeus* (*Hylaeus*) *leleji* Proshchalykin & Dathe, 2016

*Hylaeus* (*Hylaeus*) *leptocephalus* (Morawitz, 1871)

*Hylaeus* (*Hylaeus*) *nigrifacies* Bramson, 1879

*Hylaeus* (*Hylaeus*) *nigritus* (Fabricius, 1798)

*Hylaeus* (*Hylaeus*) *nimbatus* Dathe, 1986

*Hylaeus* (*Hylaeus*) *oehlkei* Dathe, 2010

*Hylaeus* (*Hylaeus*) *paulus* Bridwell, 1919

*Hylaeus* (*Hylaeus*) *pesenkoi* Proshchalykin & Dathe, 2016

*Hylaeus* (*Hylaeus*) *scutellaris* Morawitz, 1873

*Hylaeus* (*Hylaeus*) *sibiricus* (Strand, 1909)

*Hylaeus* (*Hylaeus*) *stubbei* Dathe, 1986

*Hylaeus* (*Hylaeus*) *telmenicus* Dathe, 1986

*Hylaeus* (*Hylaeus*) *tsingtauensis* (Strand, 1915)

*Hylaeus* (*Koptogaster*) *punctulatissimus* Smith, 1842

*Hylaeus* (*Lambdopsis*) *dilatatus* (Kirby, 1802)

*Hylaeus* (*Lambdopsis*) *euryscapus* Förster, 1871

*Hylaeus* (*Lambdopsis*) *pfankuchi* (Alfken, 1919)

*Hylaeus* (*Lambdopsis*) *rinki* (Gorski, 1852)

*Hylaeus* (*Nesohylaeus*) *niger* Bridwell, 1919

*Hylaeus* (*Nesoprosopis*) *floralis* (Smith, 1873)

*Hylaeus* (*Nesoprosopis*) *globulus* (Vachal, 1903)

*Hylaeus* (*Nesoprosopis*) *noomen* Hirashima, 1977

*Hylaeus* (*Nesoprosopis*) *pectoralis* Förster, 1871

*Hylaeus* (*Nesoprosopis*) *transversalis* Cockerell, 1924

*Hylaeus* (*Paraprosopis*) *clypearis* (Schenck, 1853)

*Hylaeus* (*Paraprosopis*) *concinnus* Cockerell, 1924

*Hylaeus* (*Paraprosopis*) *lineolatus* (Schenck, 1861)

*Hylaeus* (*Paraprosopis*) *pictipes* Nylander, 1852

*Hylaeus* (*Paraprosopis*) *sinuatus* (Schenck, 1853)

*Hylaeus* (*Paraprosopis*) *styriacus* Förster, 1871

*Hylaeus* (*Patagiata*) *difformis* (Eversmann, 1852)

*Hylaeus* (*Patagiata*) *nigrocuneatus* Cockerell, 1924

*Hylaeus* (*Prosopis*) *confusus* Nylander, 1852

*Hylaeus* (*Prosopis*) *gibbus* Saunders, 1850

*Hylaeus* (*Prosopis*) *hyrcanius* Dathe, 1980

*Hylaeus* (*Prosopis*) *incongruus* Förster, 1871

*Hylaeus* (*Prosopis*) *meridionalis* Förster, 1871

*Hylaeus* (*Prosopis*) *rugicollis* Morawitz, 1873

*Hylaeus* (*Prosopis*) *signatus* (Panzer, 1798)

*Hylaeus* (*Prosopis*) *variegatus* (Fabricius, 1798)

*Hylaeus* (*Spatulariella*) *hyalinatus* Smith, 1842

*Hylaeus* (*Spatulariella*) *iranicus* Dathe, 1980

*Hylaeus* (*Spatulariella*) *punctatus* (Brullé, 1832)

**Family ANDRENIDAE Latreille, 1802**

**Subfamily Andreninae Latreille, 1802**

**Tribe Andrenini Latreille, 1802**

**Genus *Andrena* Fabricius, 1775**

*Andrena* (*Aciandrena*) *aciculata* Morawitz, 1886

*Andrena* (*Aciandrena*) *chersona* Warncke, 1972

*Andrena* (*Aciandrena*) *tenuis* Morawitz, 1877

*Andrena* (*Aciandrena*) *volgensis* Osytshnjuk, 1994

*Andrena* (*Aenandrena*) *aeneiventris* Morawitz, 1872

*Andrena* (*Aenandrena*) *bisulcata* Morawitz, 1877

*Andrena* (*Aenandrena*) *hedikae* Jaeger, 1934

*Andrena* (*Aenandrena*) *hystrix* Schmiedeknecht, 1883

*Andrena* (*Andrena*) *aino* Tadauchi, Hirashima & Matsumura, 1987

*Andrena* (*Andrena*) *apicata* Smith, 1847

*Andrena* (*Andrena*) *benefica* Hirashima, 1962

*Andrena* (*Andrena*) *brevihirtiscopa* Hirashima, 1962

*Andrena* (*Andrena*) *clarkella* (Kirby, 1802)

*Andrena* (*Andrena*) *fucata* Smith, 1847

*Andrena* (*Andrena*) *fulva* (Müller, 1766)

*Andrena* (*Andrena*) *helvola* (Linnaeus, 1758)

*Andrena* (*Andrena*) *hondoica* Hirashima, 1962

*Andrena* (*Andrena*) *inconstans* Morawitz, 1877

*Andrena* (*Andrena*) *ishiharai* Hirashima, 1962

*Andrena* (*Andrena*) *kamtschatkaensis* Friese, 1914

*Andrena* (*Andrena*) *lapponica* Zetterstedt, 1838

*Andrena* (*Andrena*) *maukensis* Matsumura, 1911

*Andrena* (*Andrena*) *mitis* Schmiedeknecht, 1883

*Andrena* (*Andrena*) *nawai* Cockerell, 1913

*Andrena* (*Andrena*) *nycthemera* Imhoff, 1868

*Andrena* (*Andrena*) *praecox* (Scopoli, 1763)

*Andrena* (*Andrena*) *sakagamii* Tadauchi, Hirashima & Matsumura, 1987

*Andrena* (*Andrena*) *varians* (Kirby, 1802)

*Andrena* (*Brachyandrena*) *colletiformis* Morawitz, 1873

*Andrena* (*Brachyandrena*) *limonii* Osytshnjuk, 1983

*Andrena* (*Brachyandrena*) *pinguis* Ariana, Scheuchl, Tadauchi & Gusenleitner, 2009

*Andrena* (*Bryandrena*) *florea* Fabricius, 1793

*Andrena* (*Calomelissa*) *tsukubana* Hirashima, 1957

*Andrena* (*Campylogaster*) *chengtehensis* Yasumatsu, 1935

*Andrena* (*Campylogaster*) *erberi* Morawitz, 1871

*Andrena* (*Campylogaster*) *phaneroleuca* Cockerell, 1929

*Andrena* (*Charitandrena*) *hattorfiana* (Fabricius 1775)

*Andrena* (*Chlorandrena*) *cinereophila* Warncke, 1965

*Andrena* (*Chlorandrena*) *humilis* Imhoff, 1832

*Andrena* (*Chlorandrena*) *panurgimorpha* Mavromoustakis, 1957

*Andrena* (*Chlorandrena*) *taraxaci* Giraud, 1861

*Andrena* (*Chlorandrena*) *tricuspidata* Scheuchl, 2010

*Andrena* (*Chrysandrena*) *colonialis* Morawitz, 1886

*Andrena* (*Chrysandrena*) *fulvago* (Christ, 1791)

*Andrena* (*Chrysandrena*) *hesperia* Smith, 1853

*Andrena* (*Chrysandrena*) *khankensis* Osytshnjuk, 1995

*Andrena* (*Cnemidandrena*) *albicaudata* Hirashima, 1966

*Andrena* (*Cnemidandrena*) *denticulata* (Kirby, 1802)

*Andrena* (*Cnemidandrena*) *fuscipes* (Kirby, 1802)

*Andrena* (*Cnemidandrena*) *maetai* Hirashima, 1964

*Andrena* (*Cnemidandrena*) *nigriceps* (Kirby, 1802)

*Andrena* (*Cnemidandrena*) *rufoclypeata* Alfken, 1936

*Andrena* (*Cnemidandrena*) *simillima* Smith, 1851

*Andrena* (*Cnemidandrena*) *tridentata* (Kirby, 1802)

*Andrena* (*Cordandrena*) *cordialis* Morawitz, 1877

*Andrena* (*Cryptandrena*) *ventricosa* Dours, 1873

*Andrena* (*Euandrena*) *allosa* Warncke, 1975

*Andrena* (*Euandrena*) *asperula* Osytshnjuk, 1977

*Andrena* (*Euandrena*) *bicolor* Fabricius, 1775

*Andrena* (*Euandrena*) *capillosa* Morawitz, 1876

*Andrena* (*Euandrena*) *chrysopus* Pérez, 1903

*Andrena* (*Euandrena*) *fulvida* Schenck, 1853

*Andrena* (*Euandrena*) *glabriventris* Alfken, 1935

*Andrena* (*Euandrena*) *hebes* Pérez, 1905

*Andrena* (*Euandrena*) *khabarovi* Osytshnjuk, 1986

*Andrena* (*Euandrena*) *kudiana* Cockerell, 1924

*Andrena* (*Euandrena*) *meripes* Friese, 1922

*Andrena* (*Euandrena*) *mutini* Osytshnjuk, 1986

*Andrena* (*Euandrena*) *orientaliella* Osytshnjuk, 1986

*Andrena* (*Euandrena*) *roscipes* Alfken, 1933

*Andrena* (*Euandrena*) *rudolfae* Osytshnjuk, 1986

*Andrena* (*Euandrena*) *ruficrus* Nylander, 1848

*Andrena* (*Euandrena*) *symphyti* Schmiedeknecht, 1883

*Andrena* (*Euandrena*) *verae* Osytshnjuk, 1986

*Andrena* (*Euandrena*) *vulpecula* Kriechbaumer, 1873

*Andrena* (*Graecandrena*) *hyemala* Warncke, 1973

*Andrena* (*Graecandrena*) *impunctata* Pérez, 1895

*Andrena* (*Graecandrena*) *schwarzi* Warncke, 1975

*Andrena* (*Hamandrena*) *stepposa* Osytshnjuk, 1977

*Andrena* (*Holandrena*) *decipiens* Schenck, 1861

*Andrena* (*Holandrena*) *labialis* (Kirby, 1802)

*Andrena* (*Holandrena*) *labiatula* Osytshnjuk, 1993

*Andrena* (*Holandrena*) *valeriana* Hirashima, 1957

*Andrena* (*Holandrena*) *variabilis* Smith, 1853

*Andrena* (*Hoplandrena*) *dentata* Smith, 1879

*Andrena* (*Hoplandrena*) *ferox* Smith, 1847

*Andrena* (*Hoplandrena*) *miyamotoi* Hirashima, 1964

*Andrena* (*Hoplandrena*) *romankovae* Osytshnjuk, 1995

*Andrena* (*Hoplandrena*) *rosae* Panzer, 1801

*Andrena* (*Hoplandrena*) *scotica* Perkins, 1916

*Andrena* (*Hoplandrena*) *trimmerana* (Kirby, 1802)

*Andrena* (*Leimelissa*) *fallax* Eversmann, 1852

*Andrena* (*Lepidandrena*) *caprimulga* Warncke, 1975

*Andrena* (*Lepidandrena*) *curvungula* Thomson, 1870

*Andrena* (*Lepidandrena*) *florivaga* Eversmann, 1852

*Andrena* (*Lepidandrena*) *paucisquama* Noskiewicz, 1924

*Andrena* (*Lepidandrena*) *rufizona* Imhoff, 1834

*Andrena* (*Leucandrena*) *argentata* Smith, 1844

*Andrena* (*Leucandrena*) *barbilabris* (Kirby, 1802)

*Andrena* (*Leucandrena*) *parviceps* Kriechbaumer, 1873

*Andrena* (*Leucandrena*) *sericata* Imhoff, 1868

*Andrena* (*Leucandrena*) *ventralis* Imhoff, 1832

*Andrena* (*Limbandrena*) *limbata* Eversmann, 1852

*Andrena* (*Margandrena*) *marginata* Fabricius, 1776

*Andrena* (*Melanapis*) *fuscosa* Erichson, 1835

*Andrena* (*Melandrena*) *albopunctata* (Rossi, 1792)

*Andrena* (*Melandrena*) *assimilis* Radoszkowski, 1876

*Andrena* (*Melandrena*) *chrysopyga* Schenck, 1853

*Andrena* (*Melandrena*) *cineraria* (Linnaeus, 1758)

*Andrena* (*Melandrena*) *comta* Eversmann, 1852

*Andrena* (*Melandrena*) *cussariensis* Morawitz, 1886

*Andrena* (*Melandrena*) *flavipes* Panzer, 1799

*Andrena* (*Melandrena*) *gussakovskii* Lebedev, 1932

*Andrena* (*Melandrena*) *limata* Smith, 1853

*Andrena* (*Melandrena*) *magna* Warncke, 1965

*Andrena* (*Melandrena*) *metallescens* Cockerell, 1906

*Andrena* (*Melandrena*) *morio* Brullé, 1832

*Andrena* (*Melandrena*) *nigroaenea* (Kirby, 1802)

*Andrena* (*Melandrena*) *nitida* (Müller, 1776)

*Andrena* (*Melandrena*) *parathoracica* Hirashima, 1957

*Andrena* (*Melandrena*) *pyropygia* Kriechbaumer, 1873

*Andrena* (*Melandrena*) *senex* Eversmann, 1852

*Andrena* (*Melandrena*) *sibirica* Morawitz, 1888

*Andrena* (*Melandrena*) *stigmatica* Morawitz, 1895

*Andrena* (*Melandrena*) *thoracica* (Fabricius, 1775)

*Andrena* (*Melandrena*) *vaga* Panzer, 1799

*Andrena* (*Melandrena*) *watasei* Cockerell, 1913

*Andrena* (*Micrandrena*) *alfkenella* Perkins, 1914

*Andrena* (*Micrandrena*) *alutacea* Stöckhert, 1942

*Andrena* (*Micrandrena*) *enslinella* Stöckhert, 1924

*Andrena* (*Micrandrena*) *falsifica* Perkins, 1915

*Andrena* (*Micrandrena*) *falsificissima* Hirashima, 1966

*Andrena* (*Micrandrena*) *floricola* Eversmann, 1852

*Andrena* (*Micrandrena*) *hikosana* Hirashima, 1957

*Andrena* (*Micrandrena*) *lazoiana* Osytshnjuk, 1995

*Andrena* (*Micrandrena*) *magunta* Warncke, 1965

*Andrena* (*Micrandrena*) *minutissima* Osytshnjuk, 1995

*Andrena* (*Micrandrena*) *minutula* (Kirby, 1802)

*Andrena* (*Micrandrena*) *minutuloides* Perkins, 1914

*Andrena* (*Micrandrena*) *nana* (Kirby, 1802)

*Andrena* (*Micrandrena*) *nanaeformis* Noskiewicz, 1925

*Andrena* (*Micrandrena*) *nanula* Nylander, 1848

*Andrena* (*Micrandrena*) *niveata* Friese, 1887

*Andrena* (*Micrandrena*) *proxima* (Kirby, 1802)

*Andrena* (*Micrandrena*) *roripae* Osytshnjuk, 1993

*Andrena* (*Micrandrena*) *rugulosa* Stöckhert, 1935

*Andrena* (*Micrandrena*) *rugulosella* Osytshnjuk, 1993

*Andrena* (*Micrandrena*) *semilaevis* Pérez, 1903

*Andrena* (*Micrandrena*) *semirugosa* Cockerell, 1924

*Andrena* (*Micrandrena*) *sillata* Warncke, 1975

*Andrena* (*Micrandrena*) *stoeckhertella* Pittioni, 1948

*Andrena* (*Micrandrena*) *subopaca* Nylander, 1848

*Andrena* (*Micrandrena*) *tringa* Warncke, 1973

*Andrena* (*Nobandrena*) *anatolica* Alfken, 1935

*Andrena* (*Nobandrena*) *athenensis* Warncke, 1965

*Andrena* (*Nobandrena*) *flavobila* Warncke, 1965

*Andrena* (*Nobandrena*) *fratercula* Warncke, 1975

*Andrena* (*Nobandrena*) *nobilis* Morawitz, 1873

*Andrena* (*Notandrena*) *chrysosceles* (Kirby, 1802)

*Andrena* (*Notandrena*) *minor* (Radoszkowski, 1891)

*Andrena* (*Notandrena*) *nitidiuscula* Schenck, 1853

*Andrena* (*Notandrena*) *pallitarsis* Pérez, 1903

*Andrena* (*Notandrena*) *semiflava* Lebedev, 1932

*Andrena* (*Opandrena*) *schencki* Morawitz, 1866

*Andrena* (*Orandrena*) *oralis* Morawitz, 1876

*Andrena* (*Oreomelissa*) *coitana* (Kirby, 1802)

*Andrena* (*Oreomelissa*) *kamikochiana* Hirashima, 1963

*Andrena* (*Oreomelissa*) *media* (Radoszkowski, 1891)

*Andrena* (*Oreomelissa*) *mitakensis* Hirashima, 1963

*Andrena* (*Pallandrena*) *braunsiana* Friese, 1887

*Andrena* (*Parandrenella*) *atrata* Friese, 1887

*Andrena* (*Parandrenella*) *dentiventris* Morawitz, 1873

*Andrena* (*Parandrenella*) *figurata* Morawitz, 1866

*Andrena* (*Plastandrena*) *bimaculata* (Kirby, 1802)

*Andrena* (*Plastandrena*) *dzynnanica* Popov, 1949

*Andrena* (*Plastandrena*) *eversmanni* Radoszkowski, 1867

*Andrena* (*Plastandrena*) *khasania* Osytshnjuk, 1995

*Andrena* (*Plastandrena*) *pilipes* Fabricius, 1781

*Andrena* (*Plastandrena*) *tibialis* (Kirby, 1802)

*Andrena* (*Plastandrena*) *transbaicalica* Popov, 1949

*Andrena* (*Poecilandrena*) *fukuokensis* Hirashima, 1952

*Andrena* (*Poecilandrena*) *hybrida* Warncke, 1975

*Andrena* (*Poecilandrena*) *labiata* Fabricius, 1781

*Andrena* (*Poecilandrena*) *potentillae* Panzer, 1809

*Andrena* (*Poecilandrena*) *semirubra* Morawitz, 1875

*Andrena* (*Poecilandrena*) *sphecodimorpha* Hedicke, 1942

*Andrena* (*Poliandrena*) *caspica* Morawitz, 1886

*Andrena* (*Scitandrena*) *scita* Eversmann, 1852

*Andrena* (*Simandrena*) *combinata* (Christ, 1791)

*Andrena* (*Simandrena*) *congruens* Schmiedeknecht, 1884

*Andrena* (*Simandrena*) *dorsata* (Kirby, 1802)

*Andrena* (*Simandrena*) *kerriae* Hirashima, 1965

*Andrena* (*Simandrena*) *lepida* Schenck, 1861

*Andrena* (*Simandrena*) *nippon* Tadauchi & Hirashima, 1983

*Andrena* (*Simandrena*) *susterai* Alfken, 1914

*Andrena* (*Simandrena*) *opacifovea* Hirashima, 1952

*Andrena* (*Simandrena*) *transitoria* Morawitz, 1871

*Andrena* (*Simandrena*) *vetula* Lepeletier de Saint-Fargeau, 1841

*Andrena* (*Stenomelissa*) *halictoides* Smith, 1869

*Andrena* (*Taeniandrena*) *aberrans* Eversmann, 1852

*Andrena* (*Taeniandrena*) *afzeliella* (Kirby, 1802)

*Andrena* (*Taeniandrena*) *callopyrrha* Cockerell, 1929

*Andrena* (*Taeniandrena*) *eversmanniana* Osytshnjuk, 1994

*Andrena* (*Taeniandrena*) *ezoensis* Hirashima, 1965

*Andrena* (*Taeniandrena*) *gelriae* van der Vecht, 1927

*Andrena* (*Taeniandrena*) *intermedia* Thomson, 1870

*Andrena* (*Taeniandrena*) *lathyri* Alfken, 1899

*Andrena* (*Taeniandrena*) *ovatula* (Kirby, 1802)

*Andrena* (*Taeniandrena*) *russula* Lepeletier de Saint-Fargeau, 1841

*Andrena* (*Taeniandrena*) *wilkella* (Kirby, 1802)

*Andrena* (*Tarsandrena*) *angarensis* Cockerell, 1929

*Andrena* (*Tarsandrena*) *bonivuri* Osytshnjuk, 1984

*Andrena* (*Tarsandrena*) *ehnbergi* Morawitz, 1888

*Andrena* (*Tarsandrena*) *tarsata* Nylander, 1848

*Andrena* (*Trachandrena*) *haemorrhoa* (Fabricius, 1781)

*Andrena* (*Truncandrena*) *derbentina* Morawitz, 1886

*Andrena* (*Truncandrena*) *optata* Warncke, 1975

*Andrena* (*Truncandrena*) *truncatilabris* Morawitz, 1877

*Andrena* (*Ulandrena*) *altaica* Lebedev, 1932

*Andrena* (*Ulandrena*) *combaella* Warncke, 1966

*Andrena* (*Ulandrena*) *elegans* Giraud, 1863

*Andrena* (*Ulandrena*) *fulvitarsis* Brullé, 1832

*Andrena* (*Ulandrena*) *osychniukae* Osytshnjuk, 1977

*Andrena* (*Ulandrena*) *polita* Smith, 1847

*Andrena* (incertae sedis) *hypopolia* Schmiedeknecht, 1884

*Andrena* (incertae sedis) *incisa* Eversmann, 1852

*Andrena* (incertae sedis) *ornata* Morawitz, 1866

*Andrena* (incertae sedis) *pseudothoracica* Engel, 2005

*Andrena* (incertae sedis) *ranunculorum* Morawitz, 1877

*Andrena* (incertae sedis) *seminuda* Friese, 1896

*Andrena* (incertae sedis) *tatjanae* Osytshnjuk, 1995

**Subfamily Panurginae Leach, 1815**

**Tribe Panurgini Leach, 1815**

**Genus *Camptopoeum* Spinola, 1843**

*Camptopoeum* (*Camptopoeum*) *friesei* Mocsáry, 1894

*Camptopoeum* (*Camptopoeum*) *frontale* (Fabricius, 1804)

**Genus *Panurginus* Nylander, 1848**

*Panurginus alpotanini* Romankova & Astafurova, 2011

*Panurginus alticolus* Morawitz, 1876

*Panurginus arsenievi* Romankova & Astafurova, 2011

*Panurginus crawfordi* Cockerell, 1914

*Panurginus herzi* Morawitz, 1891

*Panurginus kropotkini* Romankova & Astafurova, 2011

*Panurginus labiatus* (Eversmann, 1852)

*Panurginus lactipennis* Friese, 1897

*Panurginus mikhno* Romankova & Astafurova, 2011

*Panurginus muraviovi* Romankova & Astafurova, 2011

*Panurginus niger* Nylander, 1848

*Panurginus romani* Aurivillius, 1914

*Panurginus sculpturatus* Morawitz, 1872

**Genus *Panurgus* Panzer, 1806**

*Panurgus* (*Panurgus*) *calcaratus* (Scopoli, 1763)

**Tribe Melliturgini Newman, 1834**

**Genus *Melitturga* Latreille, 1809**

*Melitturga* (*Melitturga*) *clavicornis* (Latreille, 1806)

*Melitturga* (*Melitturga*) *mongolica* Alfken, 1936

*Melitturga* (*Melitturga*) *praestans* Giraud, 1861

**Family HALICTIDAE Thomson, 1869**

**Subfamily Rophitinae Schenck, 1866**

**Genus *Dufourea* Lepeletier, 1841**

*Dufourea* (*Cephalictoides*) *paradoxa* (Morawitz, 1867)

*Dufourea* (*Cyprirophites*) *coeruleocephala* Morawitz, 1872

*Dufourea* (*Dufourea*) *halictula* (Nylander, 1852)

*Dufourea* (*Dufourea*) *minuta* Lepeletier, 1841

*Dufourea* (*Halictoides*) *carinata* (Popov, 1959)

*Dufourea* (*Halictoides*) *dentiventris* (Nylander, 1841)

*Dufourea* (*Halictoides*) *graeca* Ebmer, 1976

*Dufourea* (*Halictoides*) *inermis* (Nylander, 1848)

**Genus *Flavodufourea* Ebmer, 1984**

*Flavodufourea* *flavicornis* (Friese, 1914)

**Genus *Rhophitoides* Schenck, 1861**

*Rhophitoides canus* (Eversmann, 1852)

**Genus *Rophites* Spinola, 1808**

*Rophites algirus* Pérez 1895

*Rophites caucasicus* Morawitz, 1875

*Rophites clypealis* Schwammberger, 1976

*Rophites gruenwaldti* Ebmer, 1978

*Rophites hartmanni* Friese, 1902

*Rophites quinquespinosus* Spinola, 1808

**Genus *Systropha* Illiger, 1805**

*Systropha curvicornis* (Scopoli, 1770)

*Systropha planidens* Giraud, 1861

**Subfamily Nomiinae Robertson, 1904**

**Genus *Lipotriches* Gerstaecker, 1858**

*Lipotriches* (*Austronomia*) *fruhstorferi* (Pérez, 1905)

**Genus *Nomiapis* Cockerell, 1919**

*Nomiapis bispinosa* (Brullé, 1832)

*Nomiapis diversipes* (Latreille, 1806)

*Nomiapis femoralis* (Pallas, 1773)

*Nomiapis fugax* (Morawitz, 1877)

*Nomiapis mandschurica* (Hedicke, 1940)

*Nomiapis monstrosa* (Costa, 1861)

**Genus *Pseudapis* Kirby, 1900**

*Pseudapis anatolica* (Warncke, 1976)

*Pseudapis bytinski* (Warncke, 1976)

*Pseudapis elegantissima* (Popov, 1949)

**Subfamily Nomioidinae Börner, 1919**

**Genus *Ceylalictus* Strand, 1913**

*Ceylalictus* (*Ceylalictus*) *variegatus* (Olivier, 1789)

**Genus *Nomioides* Schenck, 1867**

*Nomioides* (*Nomioides*) *minutissimus* (Rossi, 1790)

*Nomioides* (*Nomioides*) *pulverosus* Handlirsch, 1888

**Subfamily Halictinae Thomson, 1869**

**Tribe Halictini Thomson, 1869**

**Genus *Halictus* Latreille, 1804**

*Halictus* (*Argalictus*) *luganicus* Blüthgen, 1936

*Halictus* (*Argalictus*) *senilis* (Eversmann, 1852)

*Halictus* (*Halictus*) *brunnescens* (Eversmann, 1852)

*Halictus* (*Halictus*) *quadricinctus* (Fabricius, 1776)

*Halictus* (*Hexataenites*) *cochlearitarsis* (Dours, 1872)

*Halictus* (*Hexataenites*) *resurgens* Nurse, 1903

*Halictus* (*Hexataenites*) *sexcinctus* (Fabricius, 1775)

*Halictus* (*Monilapis*) *adjikenticus* Blüthgen, 1923

*Halictus* (*Monilapis*) *compressus* (Walckenaer, 1802)

*Halictus* (*Monilapis*) *patellatus* Morawitz, 1873

*Halictus* (*Monilapis*) *ponticus* Blüthgen, 1934

*Halictus* (*Monilapis*) *rossicus* Ebmer, 1978

*Halictus* (*Monilapis*) *sajoi* Blüthgen, 1923

*Halictus* (*Monilapis*) *simplex* Blüthgen, 1923

*Halictus* (*Monilapis*) *tetrazonianellus* Strand, 1909

*Halictus* (*Monilapis*) *tetrazonius* (Klug, 1817)

*Halictus* (*Monilapis*) *tsingtouensis* Strand, 1910

*Halictus* (*Pachyceble*) *confusus* Smith, 1853

*Halictus* (*Pachyceble*) *gavarnicus* Pérez, 1903

*Halictus* (*Pachyceble*) *leleji* (Pesenko, 2006)

*Halictus* (*Pachyceble*) *leucaheneus* Ebmer, 1972

*Halictus* (*Pachyceble*) *transbaikalensis* Blüthgen, 1933

*Halictus* (*Pachyceble*) *tumulorum* (Linnaeus, 1758)

*Halictus* (*Platyhalictus*) *alfkenellus* Strand, 1909

*Halictus* (*Platyhalictus*) *graecus* Blüthgen, 1933

*Halictus* (*Platyhalictus*) *lussinicus* Blüthgen, 1936

*Halictus* (*Platyhalictus*) *minor* Morawitz, 1876

*Halictus* (*Protohalictus*) *hedini* Blüthgen, 1934

*Halictus* (*Protohalictus*) *rubicundus* (Christ, 1791)

*Halictus* (*Seladonia*) *aerarius* Smith, 1873

*Halictus* (*Seladonia*) *cephalicus* Morawitz, 1873

*Halictus* (*Seladonia*) *kessleri* Bramson, 1879

*Halictus* (*Seladonia*) *mondaensis* Blüthgen, 1923

*Halictus* (*Seladonia*) *mongolicus* Morawitz, 1880

*Halictus* (*Seladonia*) *nikolskayae* (Pesenko, 2006)

*Halictus* (*Seladonia*) *seladonius* (Fabricius, 1794)

*Halictus* (*Seladonia*) *semitectus* Morawitz, 1873

*Halictus* (*Seladonia*) *subauratus* (Rossi, 1792)

*Halictus* (*Seladonia*) *submediterraneus* (Pauly, 2015)

*Halictus* (*Tytthalictus*) *asperulus* Pérez, 1895

*Halictus* (*Tytthalictus*) *maculatus* Smith, 1848

*Halictus* (*Vestitohalictus*) *mucoreus* (Eversmann, 1852)

*Halictus* (*Vestitohalictus*) *pollinosus* Sichel, 1860

*Halictus* (*Vestitohalictus*) *pseudomucoreus* Ebmer, 1975

*Halictus* (*Vestitohalictus*) *pulvereus* Morawitz, 1873

*Halictus* (*Vestitohalictus*) *radoszkovskii* Vachal, 1902

*Halictus* (*Vestitohalictus*) *tectus* Radoszkowski, 1876

*Halictus* (*Vestitohalictus*) *tuberculatus* Blüthgen, 1925

**Genus *Lasioglossum* Curtis, 1833**

*Lasioglossum* (*Acanthalictus*) *dybowskii* (Radoszkowski, 1877)

*Lasioglossum* (*Biennilaeus*) *marginatum* (Brullé, 1832)

*Lasioglossum* (*Ctenonomia*) *blakistoni* Sakagami & Munakata, 1990

*Lasioglossum* (*Dialictus*) *aeratum* (Kirby, 1802)

*Lasioglossum* (*Dialictus*) *angaricum* (Cockerell, 1937)

*Lasioglossum* (*Dialictus*) *annulipes* (Morawitz, 1876)

*Lasioglossum* (*Dialictus*) *bavaricum* (Blüthgen, 1930)

*Lasioglossum* (*Dialictus*) *briseis* briseis Ebmer, 2005

*Lasioglossum* (*Dialictus*) *duckei* (Alfken, 1909)

*Lasioglossum* (*Dialictus*) *ellipticeps* (Blüthgen, 1923)

*Lasioglossum* (*Dialictus*) *gilanum* (Blüthgen, 1931)

*Lasioglossum* (*Dialictus*) *kirgisicum* Ebmer, 1972

*Lasioglossum* (*Dialictus*) *leucopum* (Kirby, 1802)

*Lasioglossum* (*Dialictus*) *lissonotum* (Noskiewicz, 1926)

*Lasioglossum* (*Dialictus*) *littorale* (Blüthgen, 1924)

*Lasioglossum* (*Dialictus*) *miyabei* Murao, Ebmer & Tadauchi, 2006

*Lasioglossum* (*Dialictus*) *morio* (Fabricius, 1793)

*Lasioglossum* (*Dialictus*) *nitidulum* (Fabricius, 1804)

*Lasioglossum* (*Dialictus*) *podolicum* (Noskiewicz, 1925)

*Lasioglossum* (*Dialictus*) *problematicum* (Blüthgen, 1923)

*Lasioglossum* (*Dialictus*) *tauricum* Ebmer, 1972

*Lasioglossum* (*Dialictus*) *virideglaucum* Ebmer & Sakagami, 1994

*Lasioglossum* (*Dialictus*) *viridellum* (Cockerell, 1931)

*Lasioglossum* (*Hemihalictus*) *adabaschum* (Blüthgen, 1931)

*Lasioglossum* (*Hemihalictus*) *allodalum* Ebmer & Sakagami, 1985

*Lasioglossum* (*Hemihalictus*) *amurense* (Vachal, 1902)

*Lasioglossum* (*Hemihalictus*) *bluethgeni* (Ebmer, 1971)

*Lasioglossum* (*Hemihalictus*) *brachycephalum* (Cockerell, 1925)

*Lasioglossum* (*Hemihalictus*) *brevicorne* (Schenck, 1869)

*Lasioglossum* (*Hemihalictus*) *buccale* (Pérez, 1903)

*Lasioglossum* (*Hemihalictus*) *clypeare* (Schenck, 1853)

*Lasioglossum* (*Hemihalictus*) *convexiusculum* (Schenck, 1853)

*Lasioglossum* (*Hemihalictus*) *corvinum* (Morawitz, 1877)

*Lasioglossum* (*Hemihalictus*) *crassepunctatum* (Blüthgen, 1923)

*Lasioglossum* (*Hemihalictus*) *elegans* (Lepeletier, 1841)

*Lasioglossum* (*Hemihalictus*) *epiphron* Ebmer, 1982

*Lasioglossum* (*Hemihalictus*) *eriphyle* Ebmer, 1996

*Lasioglossum* (*Hemihalictus*) *gorge* Ebmer, 1982

*Lasioglossum* (*Hemihalictus*) *griseolum* (Morawitz, 1872)

*Lasioglossum* (*Hemihalictus*) *intermedium* (Schenck, 1869)

*Lasioglossum* (*Hemihalictus*) *kankaucharis* (Strand, 1914)

*Lasioglossum* (*Hemihalictus*) *kiautschouense* (Strand, 1910)

*Lasioglossum* (*Hemihalictus*) *limbellum* (Morawitz, 1876)

*Lasioglossum* (*Hemihalictus*) *lucidulum* (Schenck, 1861)

*Lasioglossum* (*Hemihalictus*) *marginellum* (Schenck, 1853)

*Lasioglossum* (*Hemihalictus*) *matianense* (Blüthgen, 1926)

*Lasioglossum* (*Hemihalictus*) *medinai* (Vachal, 1895)

*Lasioglossum* (*Hemihalictus*) *mesosclerum* (Pérez, 1903)

*Lasioglossum* (*Hemihalictus*) *minutissimum* (Kirby, 1802)

*Lasioglossum* (*Hemihalictus*) *monstrificum* (Morawitz, 1891)

*Lasioglossum* (*Hemihalictus*) *nitidiusculum* (Kirby, 1802)

*Lasioglossum* (*Hemihalictus*) *pallidum* (Radoszkowski, 1888)

*Lasioglossum* (*Hemihalictus*) *pallilomum* (Strand, 1914)

*Lasioglossum* (*Hemihalictus*) *parvulum* (Schenck, 1853)

*Lasioglossum* (*Hemihalictus*) *peregrinum* (Blüthgen, 1923)

*Lasioglossum* (*Hemihalictus*) *perplexans* (Cockerell, 1925)

*Lasioglossum* (*Hemihalictus*) *punctatissimum* (Schenck, 1853)

*Lasioglossum* (*Hemihalictus*) *puncticolle* (Morawitz, 1872)

*Lasioglossum* (*Hemihalictus*) *pygmaeum* (Schenck, 1853)

*Lasioglossum* (*Hemihalictus*) *quadrinotatulum* (Schenck, 1861)

*Lasioglossum* (*Hemihalictus*) *quadrisignatum* (Schenck, 1853)

*Lasioglossum* (*Hemihalictus*) *rufitarse* (Zetterstedt, 1838)

*Lasioglossum* (*Hemihalictus*) *sakagamii* Ebmer, 1978

*Lasioglossum* (*Hemihalictus*) *samaricum* (Blüthgen, 1935)

*Lasioglossum* (*Hemihalictus*) *scirpaceum* (Warncke, 1975)

*Lasioglossum* (*Hemihalictus*) *semilaeve* (Blüthgen, 1923)

*Lasioglossum* (*Hemihalictus*) *semilucens* (Alfken, 1914)

*Lasioglossum* (*Hemihalictus*) *sexstrigatum* (Schenck, 1869)

*Lasioglossum* (*Hemihalictus*) *simplicior* (Cockerell, 1931)

*Lasioglossum* (*Hemihalictus*) *speculinum* (Cockerell, 1925)

*Lasioglossum* (*Hemihalictus*) *subaenescens* (Pérez, 1895)

*Lasioglossum* (*Hemihalictus*) *sulcatulum* (Cockerell, 1925)

*Lasioglossum* (*Hemihalictus*) *tarsatum* (Schenck, 1869)

*Lasioglossum* (*Hemihalictus*) *transpositum* (Cockerell, 1925)

*Lasioglossum* (*Hemihalictus*) *trichorhinum* (Cockerell, 1925)

*Lasioglossum* (*Hemihalictus*) *truncaticolle* (Morawitz, 1877)

*Lasioglossum* (*Hemihalictus*) *villosulum* (Kirby, 1802)

*Lasioglossum* (*Lasioglossum*) *bicallosum* (Morawitz, 1873)

*Lasioglossum* (*Lasioglossum*) *caspicum* (Morawitz, 1873)

*Lasioglossum* (*Lasioglossum*) *chloropus* (Morawitz, 1894)

*Lasioglossum* (*Lasioglossum*) *costulatum* (Kriechbaumer, 1873)

*Lasioglossum* (*Lasioglossum*) *cristula* (Pérez, 1895)

*Lasioglossum (Lasioglossum*) *eos* Ebmer, 1978

*Lasioglossum* (*Lasioglossum*) *euxinicum* Ebmer, 1972

*Lasioglossum* (*Lasioglossum*) *exiliceps* (Vachal, 1903)

*Lasioglossum* (*Lasioglossum*) *fallax* (Morawitz, 1873)

*Lasioglossum* (*Lasioglossum*) *harmandi* (Vachal, 1903)

*Lasioglossum* (*Lasioglossum*) *korbi* (Blüthgen, 1929)

*Lasioglossum* (*Lasioglossum*) *kussariense* (Blüthgen, 1925)

*Lasioglossum* (*Lasioglossum*) *laevigatum* (Kirby, 1802)

*Lasioglossum* (*Lasioglossum*) *lativentre* (Schenck, 1853)

*Lasioglossum* (*Lasioglossum*) *leviventre* (Pérez, 1905)

*Lasioglossum* (*Lasioglossum*) *pallens* (Brullé, 1932)

*Lasioglossum* (*Lasioglossum*) *proximatum* (Smith, 1879)

*Lasioglossum* (*Lasioglossum*) *quadrinotatum* (Kirby, 1802)

*Lasioglossum* (*Lasioglossum*) *rostratum* (Eversmann, 1852)

*Lasioglossum* (*Lasioglossum*) *scitulum* (Smith, 1873)

*Lasioglossum* (*Lasioglossum*) *sexnotatulum* (Nylander, 1852)

*Lasioglossum* (*Lasioglossum*) *sexnotatum* (Kirby, 1802)

*Lasioglossum* (*Lasioglossum*) *sutshanicum* Pesenko, 1986

*Lasioglossum* (*Lasioglossum*) *tungusicum* Ebmer, 1978

*Lasioglossum* (*Lasioglossum*) *xanthopus* (Kirby, 1802)

*Lasioglossum* (*Lasioglossum*) *zeyanense* Pesenko, 1986

*Lasioglossum* (*Leuchalictus*) *aegyptiellum* (Strand, 1909)

*Lasioglossum* (*Leuchalictus*) *agelastum* Fan & Ebmer, 1992

*Lasioglossum* (*Leuchalictus*) *alinense* (Cockerell, 1924)

*Lasioglossum* (*Leuchalictus*) *denticolle* (Morawitz, 1891)

*Lasioglossum* (*Leuchalictus*) *discum* (Smith, 1853)

*Lasioglossum* (*Leuchalictus*) *kansuense* (Blüthgen, 1934)

*Lasioglossum* (*Leuchalictus*) *leucozonium* (Schrank, 1781)

*Lasioglossum* (*Leuchalictus*) *majus* (Nylander, 1852)

*Lasioglossum* (*Leuchalictus*) *niveocinctum* (Blüthgen, 1923)

*Lasioglossum* (*Leuchalictus*) *occidens* (Smith, 1873)

*Lasioglossum* (*Leuchalictus*) *upinense* (Morawitz, 1889)

*Lasioglossum* (*Leuchalictus*) *zonulum* (Smith, 1848)

*Lasioglossum* (*Pyghalictus*) *glabriusculum* (Morawitz, 1872)

*Lasioglossum* (*Pyghalictus*) *kozlovi* (Friese, 1913)

*Lasioglossum* (*Pyghalictus*) *mandibulare* (Morawitz, 1866)

*Lasioglossum* (*Pyghalictus*) *politum* (Schenck, 1853)

*Lasioglossum* (*Sphecodogastra*) *affine* (Smith, 1853)

*Lasioglossum* (*Sphecodogastra*) *albipes* (Fabricius, 1781)

*Lasioglossum* (*Sphecodogastr*a) *anellum* (Vachal, 1905)

*Lasioglossum* (*Sphecodogastra*) *apristum* (Vachal, 1903)

*Lasioglossum* (*Sphecodogastra*) *baleicum* (Cockerell, 1937)

*Lasioglossum* (*Sphecodogastra*) *calceatum* (Scopoli, 1763)

*Lasioglossum* (*Sphecodogastra*) *caliginosum* Murao, Ebmer & Tadauchi, 2006

*Lasioglossum* (*Sphecodogastra*) *damascenum* (Pérez, 1911)

*Lasioglossum* (*Sphecodogastra*) *euboeense* (Strand, 1909)

*Lasioglossum* (*Sphecodogastra*) *fratellum* (Pérez, 1903)

*Lasioglossum* (*Sphecodogastra*) *fulvicorne* (Kirby, 1802)

*Lasioglossum* (*Sphecodogastra*) *hoffmanni* (Strand, 1915)

*Lasioglossum* (*Sphecodogastra*) *interruptum* (Panzer, 1798)

*Lasioglossum* (*Sphecodogastra*) *laeve* (Kirby, 1802)

*Lasioglossum* (*Sphecodogastra*) *laevoides* Ebmer, 2005

*Lasioglossum* (*Sphecodogastra*) *laticeps* laticeps (Schenck, 1869)

*Lasioglossum* (*Sphecodogastra*) *linearе* (Schenck, 1869)

*Lasioglossum* (*Sphecodogastra*) *malachurum* (Kirby, 1802)

*Lasioglossum* (*Sphecodogastra*) *minutuloides* Ebmer, 1978

*Lasioglossum* (*Sphecodogastra*) *nigripes* (Lepeletier, 1841)

*Lasioglossum* (*Sphecodogastra*) *nipponense* (Hirashima, 1953)

*Lasioglossum* (*Sphecodogastra*) *nodicorne* (Morawitz, 1889)

*Lasioglossum* (*Sphecodogastra*) *obscuratum* (Morawitz, 1876)

*Lasioglossum* (*Sphecodogastra*) *pauxillum* (Schenck, 1853)

*Lasioglossum* (*Sphecodogastra*) *setulellum* (Strand, 1909)

*Lasioglossum* (*Sphecodogastra*) *sibiriacum* (Blüthgen, 1923)

*Lasioglossum* (*Sphecodogastra*) *subfulvicorne* (Blüthgen, 1934)

*Lasioglossum* (*Sphecodogastra*) *tricinctum* (Schenck, 1874)

*Lasioglossum* (*Sphecodogastra*) *trispine* (Vachal, 1903)

*Lasioglossum* (*Sphecodogastra*) *vulsum* (Vachal, 1903)

*Lasioglossum* (*Sphecodogastra*) *yakuticum* (Pesenko & Davydova, 2004)

**Genus *Sphecodes* Latreille, 1804**

*Sphecodes* *albilabris* (Fabricius, 1793)

*Sphecodes* *alternatus* Smith, 1853

*Sphecodes* *crassus* Thomson, 1870

*Sphecodes* *cristatus* Hagens, 1882

*Sphecodes croaticus* Meyer, 1922

*Sphecodes* *ephippius* (Linnaeus, 1767)

*Sphecodes* *ferruginatus* Hagens, 1882

*Sphecodes* *geoffrellus* (Kirby, 1802)

*Sphecodes* *gibbus* (Linnaeus, 1758)

*Sphecodes* *hyalinatus* Hagens, 1882

*Sphecodes* *intermedius* Blüthgen, 1923

*Sphecodes kozlovi* Astafurova & Proshchalykin, 2015

*Sphecodes laticaudatus* Tsuneki, 1983

*Sphecodes* *longulus* Hagens, 1882

*Sphecodes majalis* Pérez, 1903

*Sphecodes* *maruyamanus* Tsuneki, 1983

*Sphecodes* *miniatus* Hagens, 1882

*Sphecodes* *monilicornis* (Kirby, 1802)

*Sphecodes* *murotai* Tsuneki, 1983

*Sphecodes* *niger* Hagens, 1874

*Sphecodes nippon* Meyer, 1922

*Sphecodes* *nomioidis* Pesenko, 1979

*Sphecodes okuyetsu* Tsuneki, 1984

*Sphecodes* *olivieri* Lepeletier de Saint Fargeau, 1825

*Sphecodes* *pellucidus* Smith, 1845

*Sphecodes* *pieli* Cockerell, 1931

*Sphecodes* *pinguiculus* Pérez, 1903

*Sphecodes* *pseudofasciatus* Blüthgen, 1924

*Sphecodes* *puncticeps* Thomson, 1870

*Sphecodes* *reticulatus* Thomson, 1870

*Sphecodes* *rubicundus* Hagens, 1875

*Sphecodes* *rufiventris* (Panzer, 1798)

*Sphecodes* *scabricollis* Wesmael, 1835

*Sphecodes* *schenckii* Hagens, 1882

*Sphecodes* *schwarzi* Astafurova & Proshchalykin, 2014

*Sphecodes* *simillimus* Smith, 1873

*Sphecodes* *spinulosus* Hagens, 1875

*Sphecodes* *tanoi* Tsuneki, 1983

**Family MELITTIDAE Schenck, 1860**

**Subfamily Dasypodainae Sagemehl, 1882**

**Tribe Dasypodaini Sagemehl, 1882**

**Genus *Dasypoda* Latreille, 1802**

*Dasypoda* (*Dasypoda*) *hirtipes* (Fabricius, 1793)

*Dasypoda* (*Dasypoda*) *japonica* Cockerell, 1911

*Dasypoda* (*Dasypoda*) *morawitzi* Radchenko, 2016

*Dasypoda* (*Megadasypoda*) *argentata* Panzer, 1809

*Dasypoda* (*Megadasypoda*) *braccata* Eversmann, 1852

*Dasypoda* (*Megadasypoda*) *spinigera* Kohl, 1905

*Dasypoda* (*Megadasypoda*) *suripes* (Christ, 1791)

*Dasypoda* (*Megadasypoda*) *toroki* Michez, 2004

**Subfamily Melittinae Schenck, 1860**

**Genus *Macropis* Panzer, 1809**

*Macropis* (*Macropis*) *dimidiata* Yasumatsu & Hirashima, 1956

*Macropis* (*Macropis*) *europaea* Warncke, 1973

*Macropis* (*Macropis*) *frivaldszkyi* Mocsáry, 1878

*Macropis* (*Macropis*) *fulvipes* (Fabricius, 1804)

*Macropis* (*Paramacropis*) *ussuriana* (Popov, 1936)

**Genus *Melitta* Kirby, 1802**

*Melitta budashkini* Radchenko & Ivanov, 2012

*Melitta budensis* (Mocsáry, 1878)

*Melitta ezoana* Yasumatsu & Hirashima, 1956

*Melitta dimidiata* Morawitz, 1875

*Melitta haemorrhoidalis* (Fabricius, 1775)

*Melitta japonica* Yasumatsu & Hirashima, 1956

*Melitta latronis* Cockerell, 1924

*Melitta leporina* (Panzer, 1799)

*Melitta melanura* (Nylander, 1852)

*Melitta nigricans* Alfken, 1905

*Melitta sibirica* (Morawitz, 1888)

*Melitta tricincta* Kirby, 1802

*Melitta udmurtica* Sitdikov, 1986

**Family MEGACHILIDAE Latreille, 1802**

**Subfamily Megachilinae Latreille, 1802**

**Tribe Lithurgini Newman, 1834**

**Genus *Lithurgus*** **Berthold, 1827**

*Lithurgus chrysurus* Fonscolombe, 1834

*Lithurgus cornutus* (Fabricius, 1787)

*Lithurgus tibialis* Morawitz, 1875

**Tribe Osmiini Newman, 1834**

**Genus *Chelostoma* Latreille, 1809**

*Chelostoma* (*Chelostoma*) *florisomne* (Linnaeus, 1758)

*Chelostoma* (*Chelostoma*) *mocsaryi* Schletterer, 1889

*Chelostoma* (*Foveosmia*) *campanularum* (Kirby, 1802)

*Chelostoma* (*Foveosmia*) *distinctum* (Stöckhert, 1929)

*Chelostoma* (*Foveosmia*) *foveolatum* (Morawitz, 1868)

*Chelostoma* (*Gyrodromella*) *rapunculi* (Lepeletier de Saint-Fargeau, 1841)

**Genus *Heriades* Spinola, 1808**

*Heriades* (*Heriades*) *crenulata* Nylander, 1856

*Heriades* (*Heriades*) *rubicola* Pérez, 1890

*Heriades* (*Heriades*) *truncorum* (Linnaeus, 1758)

**Genus *Hoplitis* Klug, 1807**

*Hoplitis* (*Alcidamea*) *acuticornis* (Dufour & Perris, 1840)

*Hoplitis* (*Alcidamea*) *beijingensis* Wu, 1987

*Hoplitis* (*Alcidamea*) *claviventris* (Thomson, 1872)

*Hoplitis* (*Alcidamea*) *curvipes* (Morawitz, 1871)

*Hoplitis* (*Alcidamea*) *fulva* (Eversmann, 1852)

*Hoplitis* (*Alcidamea*) *leucomelana* (Kirby, 1802)

*Hoplitis* (*Alcidamea*) *mitis* (Nylander, 1852)

*Hoplitis* (*Alcidamea*) *mollis* Tkalců, 2000

*Hoplitis* (*Alcidamea*) *ozbeki* Tkalců, 2000

*Hoplitis* (*Alcidamea*) *praestans* (Morawitz, 1893)

*Hoplitis* (*Alcidamea*) *princeps* (Morawitz, 1872)

*Hoplitis* (*Alcidamea*) *scita* (Eversmann, 1852)

*Hoplitis* (*Alcidamea*) *tridentata* (Dufour & Perris, 1840)

*Hoplitis* (*Alcidamea*) *tuberculata* (Nylander, 1848)

*Hoplitis* (*Anthocopa*) *caucasicola* Müller, 2012

*Hoplitis* (*Anthocopa*) *daurica* (Radoszkowski, 1887)

*Hoplitis* (*Anthocopa*) *jakovlevi* (Radoszkowski, 1874)

*Hoplitis* (*Anthocopa*) *mocsaryi* (Friese, 1895)

*Hoplitis* (*Anthocopa*) *papaveris* (Latreille, 1799)

*Hoplitis* (*Anthocopa*) *saxialis* (van der Zanden, 1994)

*Hoplitis* (*Anthocopa*) *villosa* (Schenck, 1853)

*Hoplitis* (*Formicapis*) *maritima* (Romankova, 1985)

*Hoplitis* (*Formicapis*) *robusta* (Nylander, 1848)

*Hoplitis* (*Hoplitis*) *adunca* (Panzer, 1798)

*Hoplitis* (*Hoplitis*) *anthocopoides* (Schenck, 1853)

*Hoplitis* (*Hoplitis*) *astragali* Fateryga, Müller & Proshchalykin, 2023

*Hoplitis* (*Hoplitis*) *carinata* (Stanek, 1969)

*Hoplitis* (*Hoplitis*) *dagestanica* Fateryga, Müller & Proshchalykin, 2023

*Hoplitis* (*Hoplitis*) *kaszabi* Tkalců, 2000

*Hoplitis* (*Hoplitis*) *linguaria* (Morawitz, 1875)

*Hoplitis* (*Hoplitis*) *manicata* Morice, 1901

*Hoplitis* (*Pentadentosmia*) *laevifrons* (Morawitz, 1872)

*Hoplitis* (*Platosmia*) *inconspicua* Tkalců, 1995

**Genus *Osmia* Panzer, 1806**

*Osmia* (*Allosmia*) *rufohirta* Latreille, 1811

*Osmia* (*Erythrosmia*) *andrenoides* Spinola, 1808

*Osmia* (*Helicosmia*) *aurulenta* (Panzer, 1799)

*Osmia* (*Helicosmia*) *caerulescens* (Linnaeus, 1758)

*Osmia* (*Helicosmia*) *cinerea* Warncke, 1988

*Osmia* (*Helicosmia*) *dimidiata* Morawitz, 1870

*Osmia* (*Helicosmia*) *leaiana* (Kirby, 1802)

*Osmia* (*Helicosmia*) *melanogaster* Spinola, 1808

*Osmia* (*Helicosmia*) *niveata* (Fabricius, 1804)

*Osmia* (*Helicosmia*) *orientalis* Benoist, 1929

*Osmia* (*Helicosmia*) *signata* Erichson, 1835

*Osmia* (*Hoplosmia*) *bidentata* Morawitz, 1875

*Osmia* (*Hoplosmia*) *ligurica* Morawitz, 1868

*Osmia* (*Hoplosmia*) *scutellaris* Morawitz, 1868

*Osmia* (*Hoplosmia*) *spinulosa* (Kirby, 1802)

*Osmia* (*Melanosmia*) *disjuncta* Tkalců, 1995

*Osmia* (*Melanosmia*) *ephippiata* Smith, 1879

*Osmia* (*Melanosmia*) *inermis* (Zetterstedt, 1838)

*Osmia* (*Melanosmia*) *laticeps* Thomson, 1872

*Osmia* (*Melanosmia*) *maritima* Friese, 1885

*Osmia* (*Melanosmia*) *nigriventris* (Zetterstedt, 1838)

*Osmia* (*Melanosmia*) *parietina* Curtis, 1828

*Osmia* (*Melanosmia*) *pilicornis* Smith, 1846

*Osmia* (*Melanosmia*) *uncinata* Gerstäcker, 1869

*Osmia* (*Melanosmia*) *xanthomelana* (Kirby, 1802)

*Osmia* (*Metallinella*) *brevicornis* (Fabricius, 1798)

*Osmia* (*Neosmia*) *bicolor* (Schrank, 1781)

*Osmia* (*Osmia*) *apicata* Smith, 1853

*Osmia* (*Osmia*) *bicornis* (Linnaeus, 1758)

*Osmia* (*Osmia*) *cerinthidis* Morawitz, 1876

*Osmia* (*Osmia*) *cornifrons* (Radoszkowski, 1887)

*Osmia* (*Osmia*) *cornuta* (Latreille, 1805)

*Osmia* (*Osmia*) *mustelina* Gerstäcker, 1869

*Osmia* (*Osmia*) *opima* Romankova, 1985

*Osmia* (*Osmia*) *pedicornis* Cockerell, 1920

*Osmia* (*Osmia*) *taurus* Smith, 1873

*Osmia* (*Pyrosmia*) *cephalotes* Morawitz, 1870

*Osmia* (*Pyrosmia*) *cyanoxantha* Pérez, 1879

*Osmia* (*Pyrosmia*) *hellados* van der Zanden, 1984

*Osmia* (*Pyrosmia*) *nana* Morawitz, 1873

*Osmia* (*Pyrosmia*) *submicans* Morawitz, 1870

*Osmia* (*Pyrosmia*) *versicolor* Latreille, 1811

*Osmia* (*Pyrosmia*) *viridana* Morawitz, 1873

*Osmia* (*Tergosmia*) *tergestensis* Ducke, 1897

**Genus *Protosmia*** **Ducke, 1900**

*Protosmia* (*Protosmia*) *glutinosa* (Giraud, 1871)

*Protosmia* (*Protosmia*) *tauricola* Popov, 1961

*Protosmia* (*Protosmia*) *tiflensis* (Morawitz, 1876)

**Tribe Anthidiini Ashmead, 1899**

**Genus *Anthidiellum* Cockerell, 1904**

*Anthidiellum* (*Anthidiellum*) *strigatum* (Panzer, 1805)

*Anthidiellum* (*Anthidiellum*) *troodicum* Mavromoustakis, 1949

**Genus *Anthidium* Fabricius, 1804**

*Anthidium* (*Anthidium*) *amurense* Radoszkowski, 1876

*Anthidium* (*Anthidium*) *cingulatum* Latreille, 1809

*Anthidium* (*Anthidium*) *dalmaticum* Mocsáry, 1884

*Anthidium* (*Anthidium*) *diadema* Latreille, 1809

*Anthidium* (*Anthidium*) *florentinum* (Fabricius, 1775)

*Anthidium* (*Anthidium*) *loti* Perris, 1852

*Anthidium* (*Anthidium*) *manicatum* (Linnaeus, 1758)

*Anthidium* (*Anthidium*) *melanopygum* Friese, 1917

*Anthidium* (*Anthidium*) *montanum* Morawitz, 1865

*Anthidium* (*Anthidium*) *punctatum* Latreille, 1809

*Anthidium* (*Anthidium*) *septemspinosum* Lepeletier de Saint-Fargeau, 1841

*Anthidium* (*Proanthidium*) *fulviventre* Friese, 1917

*Anthidium* (*Proanthidium*) *oblongatum* (Illiger, 1806)

**Genus *Bathanthidium* Mavromoustakis, 1953**

*Bathanthidium* (*Stenanthidiellum*) *malaisei* (Popov, 1941)

*Bathanthidium* (*Stenanthidiellum*) *sibiricum* (Eversmann, 1852)

**Genus *Eoanthidium* Popov, 1950**

*Eoanthidium* (*Eoanthidium*) *clypeare* (Morawitz, 1873)

**Genus *Icteranthidium* Michener, 1948**

*Icteranthidium fedtschenkoi* (Morawitz, 1875)

*Icteranthidium ferrugineum* (Fabricius, 1787)

*Icteranthidium floripetum* (Eversmann, 1852)

*Icteranthidium grohmanni* (Spinola, 1838)

*Icteranthidium laterale* (Latreille, 1809)

**Genus *Pseudoanthidium*** **Friese, 1898**

*Pseudoanthidium* (*Exanthidium*) *eximium* (Giraud, 1863)

*Pseudoanthidium* (*Pseudoanthidium*) *alpinum* (Morawitz, 1873)

*Pseudoanthidium* (*Pseudoanthidium*) *nanum* (Mocsáry, 1880)

*Pseudoanthidium* (*Pseudoanthidium*) *stigmaticorne* (Dours, 1873)

*Pseudoanthidium* (*Pseudoanthidium*) *tenellum* (Mocsáry, 1880)

*Pseudoanthidium* (*Royanthidium*) *melanurum* (Klug, 1832)

*Pseudoanthidium* (*Royanthidium*) *reticulatum* (Mocsáry, 1884)

**Genus *Stelis* Panzer, 1806**

*Stelis* (*Heterostelis*) *annulata* (Lepeletier de Saint-Fargeau, 1841)

*Stelis* (*Protostelis*) *signata* (Latreille, 1809)

*Stelis* (*Pseudostelis*) *minima* Schenck, 1861

*Stelis* (*Pseudostelis*) *minuta* Lepeletier de Saint-Fargeau & Audinet-Serville, 1825

*Stelis* (*Stelidomorpha*) *nasuta* (Latreille, 1809)

*Stelis* (*Stelis*) *aculeata* Morawitz, 1880

*Stelis* (*Stelis*) *breviuscula* (Nylander, 1848)

*Stelis* (*Stelis*) *melanura* Cockerell, 1924

*Stelis* (*Stelis*) *odontopyga* Noskiewicz, 1926

*Stelis* (*Stelis*) *ornatula* (Klug, 1807)

*Stelis* (*Stelis*) *phaeoptera* (Kirby, 1802)

*Stelis* (*Stelis*) *punctulatissima* (Kirby, 1802)

*Stelis* (*Stelis*) *scutellaris* Morawitz, 1894

*Stelis* (*Stelis*) *simillima* Morawitz, 1875

**Genus *Trachusa* Panzer, 1804**

*Trachusa* (*Archianthidium*) *pubescens* (Morawitz, 1872)

*Trachusa* (*Paraanthidium*) *integra* (Eversmann, 1852)

*Trachusa* (*Trachusa*) *byssina* (Panzer, 1798)

**Tribe Dioxyini Cockerell, 1902**

**Genus *Aglaoapis* Cameron, 1901**

*Aglaoapis tridentata* (Nylander, 1848)

**Genus *Dioxys*** **Lepeletier de Saint-Fargeau & Audinet-Serville, 1825**

*Dioxys cinctus* (Jurine, 1807)

**Tribe Megachilini Latreille, 1802**

**Genus *Coelioxys* Latreille, 1809**

*Coelioxys* (*Allocoelioxys*) *acanthura* (Illiger, 1806)

*Coelioxys* (*Allocoelioxys*) *afer* Lepeletier de Saint-Fargeau, 1841

*Coelioxys* (*Allocoelioxys*) *argenteus* Lepeletier de Saint-Fargeau, 1841

*Coelioxys* (*Allocoelioxys*) *brevis* Eversmann, 1852

*Coelioxys* (*Allocoelioxys*) *caudatus* Spinola, 1838

*Coelioxys* (*Allocoelioxys*) *echinatus* Förster, 1853

*Coelioxys* (*Allocoelioxys*) *elsei* Schwarz, 2001

*Coelioxys* (*Allocoelioxys*) *emarginatus* Förster, 1853

*Coelioxys* (*Allocoelioxys*) *formosicola* Strand, 1913

*Coelioxys* (*Allocoelioxys*) *haemorrhoa* Förster, 1853

*Coelioxys* (*Allocoelioxys*) *mielbergi* Morawitz, 1880

*Coelioxys* (*Allocoelioxys*) *obtusus* Pérez, 1884

*Coelioxys* (*Allocoelioxys*) *polycentris* Förster, 1853

*Coelioxys* (*Coelioxys*) *quadridentatus* (Linnaeus, 1758)

*Coelioxys* (*Liothyrapis*) *decipiens* (Spinola, 1838)

*Coelioxys* (*Melissoctonia*) *conoideus* (Illiger, 1806)

*Coelioxys* (*Paracoelioxys*) *alatus* Förster, 1853

*Coelioxys* (*Paracoelioxys*) *elongatus* Lepeletier de Saint-Fargeau, 1841

*Coelioxys* (*Paracoelioxys*) *inermis* (Kirby, 1802)

*Coelioxys* (*Paracoelioxys*) *mandibularis* Nylander, 1848

*Coelioxys* (*Paracoelioxys*) *pielianus* Friese, 1935

*Coelioxys* (*Rozeniana*) *aurolimbatus* Förster, 1853

*Coelioxys* (*Rozeniana*) *rufescens* Lepeletier de Saint-Fargeau & Audinet-Serville, 1825

*Coelioxys* (*Rozeniana*) *ruficinctus* Cockerell, 1931

*Coelioxys* (incertae sedis) *lanceolatus* Nylander, 1852

*Coelioxys* (incertae sedis) *obtusispina* Thomson, 1872

**Genus *Megachile* Latreille, 1802**

*Megachile* (*Aethomegachile*) *remota* Smith, 1879

*Megachile* (*Callomegachile*) *sculpturalis* Smith, 1853

*Megachile* (*Chalicodoma*) *albocristata* Smith, 1853

*Megachile* (*Chalicodoma*) *albonotata* Radoszkowski, 1886

*Megachile* (*Chalicodoma*) *alborufa* Friese, 1911

*Megachile* (*Chalicodoma*) *desertorum* Morawitz, 1875

*Megachile* (*Chalicodoma*) *parietina* (Geoffroy, 1785)

*Megachile* (*Creightonella*) *albisecta* (Klug, 1817)

*Megachile* (*Eutricharaea*) *anatolica* Rebmann, 1968

*Megachile* (*Eutricharaea*) *apicalis* Spinola, 1808

*Megachile* (*Eutricharaea*) *argentata* (Fabricius, 1793)

*Megachile* (*Eutricharaea*) *burdigalensis* Benoist, 1940

*Megachile* (*Eutricharaea*) *deceptoria* Pérez, 1890

*Megachile* (*Eutricharaea*) *giraudi* Gerstäcker, 1869

*Megachile* (*Eutricharaea*) *leachella* Curtis, 1828

*Megachile* (*Eutricharaea*) *leucomalla* Gerstäcker, 1869

*Megachile* (*Eutricharaea*) *marginata* Smith, 1853

*Megachile* (*Eutricharaea*) *melanogaster* Eversmann, 1852

*Megachile* (*Eutricharaea*) *rotundata* (Fabricius, 1787)

*Megachile* (*Eutricharaea*) *rubrimana* Morawitz, 1893

*Megachile* (*Eutricharaea*) *semicircularis* auct. nec van der Zanden, 1996

*Megachile* (*Megachile*) *alpicola* Alfken, 1924

*Megachile* (*Megachile*) *bombycina* Radoszkowski, 1874

*Megachile* (*Megachile*) *centuncularis* (Linnaeus, 1758)

*Megachile* (*Megachile*) *dacica* Mocsáry, 1879

*Megachile* (*Megachile*) *genalis* Morawitz, 1880

*Megachile* (*Megachile*) *lapponica* Thomson, 1872

*Megachile* (*Megachile*) *ligniseca* (Kirby, 1802)

*Megachile* (*Megachile*) *manipula* Romankova, 1983

*Megachile* (*Megachile*) *melanopyga* Costa, 1863

*Megachile* (*Megachile*) *nipponica* Cockerell, 1914

*Megachile* (*Megachile*) *octosignata* Nylander, 1852

*Megachile* (*Megachile*) *pilicrus* Morawitz, 1877

*Megachile* (*Megachile*) *pyrenaea* Pérez, 1890

*Megachile* (*Megachile*) *versicolor* Smith, 1844

*Megachile* (*Pseudomegachile*) *ericetorum* Lepeletier de Saint-Fargeau, 1841

*Megachile* (*Pseudomegachile*) *flavipes* Spinola, 1838

*Megachile* (*Pseudomegachile*) *saussurei* Radoszkowski, 1874

*Megachile* (*Pseudomegachile*) *tecta* Radoszkowski, 1888

*Megachile* (*Xanthosarus*) *analis* Nylander, 1852

*Megachile* (*Xanthosarus*) *circumcincta* (Kirby, 1802)

*Megachile* (*Xanthosarus*) *fulvimana* Eversmann, 1852

*Megachile* (*Xanthosarus*) *lagopoda* (Linnaeus, 1761)

*Megachile* (*Xanthosarus*) *maackii* Radoszkowski, 1874

*Megachile* (*Xanthosarus*) *maritima* (Kirby, 1802)

*Megachile* (*Xanthosarus*) *nigriventris* Schenck, 1870

*Megachile* (*Xanthosarus*) *willughbiella* (Kirby, 1802)

*Megachile* (incertae sedis) *epovae* Cockerell, 1928

*Megachile* (incertae sedis) *ferritincta* Cockerell, 1924

*Megachile* (incertae sedis) *lucidula* Mocsáry, 1901

*Megachile* (incertae sedis) *polita* Cockerell, 1924

*Megachile* (incertae sedis) *scheviakovi* Cockerell, 1928

*Megachile* (incertae sedis) *sichotana* Cockerell, 1924

**Family APIDAE Latreille, 1802**

**Subfamily Xylocopinae Latreille, 1802**

**Tribe Xylocopini Latreille, 1802**

**Genus *Xylocopa* Latreille, 1802**

*Xylocopa* (*Alloxylocopa*) *appendiculata* Smith, 1852

*Xylocopa* (*Copoxyla*) *iris* (Christ, 1791)

*Xylocopa* (*Proxylocopa*) *olivieri* Lepeletier de Saint-Fargeau, 1841

*Xylocopa* (*Proxylocopa*) *altaica* Popov, 1947

*Xylocopa* (*Xylocopa*) *valga* Gerstäcker, 1872

*Xylocopa* (*Xylocopa*) *violacea* (Linnaeus, 1758)

**Tribe Ceratinini Latreille, 1802**

**Genus *Ceratina* Latreille, 1802**

*Ceratina* (*Ceratina*) *cucurbitina* (Rossi, 1792)

*Ceratina* (*Ceratina*) *satoi* Yasumatsu, 1936

*Ceratina* (*Ceratinidia*) *flavipes* Smith, 1879

*Ceratina* (*Euceratina*) *acuta* Friese, 1896

*Ceratina* (*Euceratina*) *cyanea* (Kirby 1802)

*Ceratina* (*Euceratina*) *chalcites* Germar, 1839

*Ceratina* (*Euceratina*) *chalybea* Chevrier, 1872

*Ceratina* (*Euceratina*) *chrysomalla* Gerstäcker, 1869

*Ceratina* (*Euceratina*) *dallatorreana* Friese, 1896

*Ceratina* (*Euceratina*) *gravidula* Gerstäcker, 1869

*Ceratina* (*Euceratina*) *laevifrons* Morawitz 1894

*Ceratina* (*Euceratina*) *nigroaenea* Gerstäcker, 1869

*Ceratina* (*Euceratina*) *nigrolabiata* Friese, 1896

*Ceratina* (*Euceratina*) *zwakhalsi* Terzo & Rasmont, 1997

**Subfamily Nomadinae Latreille, 1802**

**Tribe Nomadini Latreille, 1802**

**Genus *Nomada* Scopoli, 1770**

*Nomada albidemaculata* Łoziński, 1922

*Nomada alboguttata* Herrich-Schäffer, 1839

*Nomada amurensis* Radoszkowski, 1876

*Nomada argentata* Herrich-Schäffer, 1839

*Nomada armata* Herrich-Schäffer, 1839

*Nomada arrogans* Schmiedeknecht, 1882

*Nomada atroscutellaris* Strand, 1921

*Nomada basalis* Herrich-Schäffer, 1839

*Nomada bifasciata* Olivier, 1811

*Nomada bispinosa* Mocsáry, 1883

*Nomada blepharipes* Schmiedeknecht, 1882

*Nomada bluethgeni* Stöckhert, 1944

*Nomada braunsiana* Schmiedeknecht, 1882

*Nomada calimorpha* Schmiedeknecht, 1882

*Nomada castellana* Dusmet, 1913

*Nomada comparata* Cockerell, 1911

*Nomada conjungens* Herrich-Schäffer, 1839

*Nomada corcyraea* Schmiedeknecht, 1882

*Nomada coxalis* Morawitz, 1877

*Nomada cruenta* Schmiedeknecht, 1882

*Nomada diacantha* Schwarz, 1981

*Nomada distinguenda* Morawitz, 1873

*Nomada dybovskij* Radoszkowski, 1876

*Nomada ecarinata* Morawitz, 1888

*Nomada emarginata* Morawitz, 1877

*Nomada errans* Lepeletier de Saint-Fargeau, 1841

*Nomada erythrocephala* Morawitz, 1871

*Nomada esana* Tsuneki, 1973

*Nomada fabriciana* (Linnaeus, 1767)

*Nomada facilis* Schwarz, 1967

*Nomada femoralis* Morawitz, 1868

*Nomada ferruginata* (Linnaeus, 1767)

*Nomada flava* Panzer, 1798

*Nomada flavoguttata* (Kirby, 1802)

*Nomada flavopicta* (Kirby, 1802)

*Nomada fucata* Panzer, 1798

*Nomada fulvicornis* Fabricius, 1793

*Nomada furva* Panzer, 1798

*Nomada furvoides* Stöckhert, 1944

*Nomada fusca* Schwarz, 1986

*Nomada fuscicornis* Nylander, 1848

*Nomada ginran* Tsuneki, 1973

*Nomada glaberrima* Schmiedeknecht, 1882

*Nomada glaucopis* Pérez, 1890

*Nomada goodeniana* (Kirby, 1802)

*Nomada guttulata* Schenck, 1861

*Nomada hakusana* Tsuneki, 1973

*Nomada hammarstroemi* Morawitz, 1888

*Nomada hera* Schwarz, 1965

*Nomada hungarica* Dalla Torre & Friese, 1894

*Nomada immaculata* Morawitz, 1873

*Nomada imperialis* Schmiedeknecht, 1882

*Nomada incisa* Schmiedeknecht, 1882

*Nomada integra* Brullé, 1832

*Nomada irkutskiensis* Proshchalykin & Schwarz, 2017

*Nomada italica* Dalla Torre & Friese, 1894

*Nomada issikii* Yasumatsu, 1939

*Nomada japonica* Smith, 1873

*Nomada kohli* Schmiedeknecht, 1882

*Nomada koikensis* Tsuneki, 1973

*Nomada lathburiana* (Kirby, 1802)

*Nomada laticrus* Mocsáry, 1883

*Nomada leucophthalma* (Kirby, 1802)

*Nomada lutea* Eversmann, 1852

*Nomada maculifrons* Smith, 1869

*Nomada margelanica* Schwarz, 1987

*Nomada marshamella* (Kirby, 1802)

*Nomada mauritanica* Lepeletier de Saint-Fargeau, 1841

*Nomada minuscula* Noskiewicz, 1930

*Nomada mitaii* Proshchalykin, 2010

*Nomada mocsaryi* Schmiedeknecht, 1882

*Nomada moeschleri* Alfken, 1913

*Nomada mutabilis* Morawitz, 1871

*Nomada mutica* Morawitz, 1872

*Nomada nobilis* Herrich-Schäffer, 1839

*Nomada noskiewiczi* Schwarz, 1966

*Nomada obscura* Zetterstedt, 1838

*Nomada obtusifrons* Nylander, 1848

*Nomada oculata* Friese, 1821

*Nomada okamotonis* Matsumura, 1912

*Nomada opaca* Alfken, 1913

*Nomada pacifica* Tsuneki, 1973

*Nomada pallispinosa* Schwarz, 1967

*Nomada palmeni* Morawitz, 1888

*Nomada panzeri* Lepeletier de Saint-Fargeau, 1841

*Nomada pastoralis* Eversmann, 1852

*Nomada pectoralis* Morawitz, 1877

*Nomada piccioliana* Magretti, 1883

*Nomada piliventris* Morawitz, 1877

*Nomada pilosa* Schwarz & Gusenleitner, 2017

*Nomada posthuma* Blüthgen, 1949

*Nomada pulchra* Arnold, 1888

*Nomada pygidialis* Schwarz, 1981

*Nomada radoszkowskii* Łoziński, 1922

*Nomada rhenana* Morawitz, 1872

*Nomada roberjeotiana* Panzer, 1799

*Nomada rubricollis* Schwarz, 1966

*Nomada rubricosa* Eversmann, 1852

*Nomada ruficornis* (Linnaeus, 1758)

*Nomada rufipes* Fabricius, 1793

*Nomada setteri* Proshchalykin, 2010

*Nomada sexfasciata* Panzer, 1799

*Nomada sheppardana* (Kirby, 1802)

*Nomada silvicola* Tsuneki, 1973

*Nomada stigma* Fabricius, 1804

*Nomada striata* Fabricius, 1793

*Nomada stoeckherti* Pittioni, 1951

*Nomada subcornuta* (Kirby, 1802)

*Nomada succincta* Panzer, 1798

*Nomada sybarita* Schmiedeknecht, 1882

*Nomada tenella* Mocsáry, 1883

*Nomada thersites* Schmiedeknecht 1882

*Nomada trapeziformis* Schmiedeknecht, 1882

*Nomada tridentirostris* Dours, 1873

*Nomada trispinosa* Schmiedeknecht, 1882

*Nomada yarrowi* Schwarz, 1981

*Nomada zonata* Panzer, 1798

**Tribe Epeolini Robertson, 1903**

**Genus *Epeolus* Latreille, 1802**

*Epeolus alpinus* Friese, 1893

*Epeolus asiaticus* Astafurova & Proshchalykin, 2022

*Epeolus coreanus* Yasumatsu, 1933

*Epeolus cruciger* (Panzer, 1799)

*Epeolus fasciatus* Friese, 1895

*Epeolus julliani* Pérez, 1884

*Epeolus laticauda* Bischoff, 1930

*Epeolus melectiformis* Yasumatsu, 1938

*Epeolus mongolicus* Astafurova & Proshchalykin, 2021

*Epeolus nudiventris* Bischoff, 1930

*Epeolus productulus* Bischoff, 1930

*Epeolus rasmonti* Astafurova & Proshchalykin, 2022

*Epeolus ruficornis* Morawitz, 1875

*Epeolus schummeli* Schilling, 1849

*Epeolus tarsalis* Morawitz, 1873

*Epeolus transitorius* Eversmann, 1852

*Epeolus variegatus* (Linnaeus, 1758)

**Genus *Triepeolus* Robertson, 1901**

*Triepeolus tristis* (Smith, 1854)

*Triepeolus ventralis* (Meade-Waldo, 1913)

**Tribe Ammobatoidini Michener, 1944**

**Genus *Ammobatoides* Radoszkowski, 1867**

*Ammobatoides abdominalis* (Eversmann, 1852)

*Ammobatoides radoszkowskii* Proshchalykin & Lelej, 2014

**Tribe Biastini Linsley & Michener, 1939**

**Genus *Biastes* Panzer, 1806**

*Biastes brevicornis* (Panzer, 1798)

*Biastes emarginatus* (Schenck, 1853)

*Biastes popovi* Proshchalykin & Lelej, 2004

*Biastes truncatus* (Nylander, 1848)

**Tribe Ammobatini Handlirsch, 1925**

**Genus *Ammobates* Latreille, 1809**

*Ammobates* (*Ammobates*) *opacus* Popov, 1951

*Ammobates* (*Ammobates*) *punctatus* (Fabricius 1804)

*Ammobates* (*Ammobates*) *vinctus* Gerstäcker, 1869

*Ammobates* (*Euphileremus*) *oraniensis* (Lepeletier de Saint-Fargeau, 1841)

**Genus *Parammobatodes* Popov, 1931**

*Parammobatodes minutus* (Mocsáry, 1878)

**Genus *Pasites* Jurine, 1807**

*Pasites esakii* Popov & Yasumatsu, 1935

*Pasites maculatus* Jurine, 1807

**Subfamily Apinae Latreille, 1802**

**Tribe Osirini Handlirsch, 1925**

**Genus *Epeoloides* Giraud, 1863**

*Epeoloides coecutiens* (Fabricius, 1775)

**Tribe Ancylaini Michener, 1944**

**Genus *Ancyla* Lepeletier** **de Saint-Fargeau, 1841**

*Ancyla asiatica* Friese, 1922

**Tribe Ctenoplectrini Cockerell, 1930**

**Genus *Ctenoplectra* Kirby, 1826**

*Ctenoplectra davidi* Vachal, 1903

**Tribe Eucerini Latreille, 1802**

**Genus *Eucera* Scopoli, 1770**

*Eucera* (*Cubitalia*) *morio* Friese, 1911

*Eucera* (*Cubitalia*) *parvicornis* Mocsáry, 1878

*Eucera* (*Cubitalia*) *tristis* Morawitz, 1875

*Eucera* (*Eucera*) *albofasciata* Friese, 1895

*Eucera* (*Eucera*) *caspica* Morawitz, 1873

*Eucera* (*Eucera*) *cineraria* Eversmann, 1852

*Eucera* (*Eucera*) *clypeata* Erichson, 1835

*Eucera* (*Eucera*) *curvitarsis* Mocsáry, 1879

*Eucera* (*Eucera*) *dalmatica* Lepeletier de Saint-Fargeau, 1841

*Eucera* (*Eucera*) *excisa* Mocsáry, 1879

*Eucera* (*Eucera*) *interrupta* Baer, 1850

*Eucera* (*Eucera*) *kullenbergi* Tkalců, 1984

*Eucera* (*Eucera*) *longicornis* (Linnaeus, 1758)

*Eucera* (*Eucera*) *nigrescens* Pérez, 1879

*Eucera* (*Eucera*) *nigrifacies* Lepeletier de Saint-Fargeau, 1841

*Eucera* (*Eucera*) *nigrilabris* Lepeletier de Saint-Fargeau, 1841

*Eucera* (*Eucera*) *pannonica* Mocsáry, 1878

*Eucera* (*Eucera*) *paraclypeata* Sitdikov, 1988

*Eucera* (*Eucera*) *pollinosa* Smith, 1854

*Eucera* (*Eucera*) *proxima* Morawitz, 1875

*Eucera* (*Eucera*) *rufipes* Smith, 1879

*Eucera* (*Eucera*) *seminuda* Brullé, 1832

*Eucera* (*Eucera*) *sociabilis* Smith, 1873

*Eucera* (*Eucera*) *sogdiana* Morawitz, 1875

*Eucera* (*Eucera*) *taurica* Morawitz, 1870

*Eucera* (*Eucera*) *vittulata* Noskiewicz, 1934

*Eucera* (*Hetereucera*) *atriceps* Morawitz, 1878

*Eucera* (*Hetereucera*) *ferghanica* Morawitz, 1875

*Eucera* (*Synhalonia*) *armeniaca* (Morawitz, 1877)

*Eucera* (*Synhalonia*) *chinensis* (Smith, 1854)

*Eucera* (*Synhalonia*) *distinguenda* (Morawitz, 1875)

*Eucera* (*Synhalonia*) *hungarica* Friese, 1895

*Eucera* (*Synhalonia*) *rufa* (Lepeletier de Saint-Fargeau, 1841)

*Eucera* (*Synhalonia*) *ruficollis* (Brullé, 1832)

*Eucera* (*Synhalonia*) *tricincta* Erichson, 1835

*Eucera* (*Synhalonia*) *velutina* (Morawitz, 1873)

**Genus *Tetralonia* Spinola, 1839**

*Tetralonia alticincta* (Lepeletier de Saint-Fargeau, 1841)

*Tetralonia dentata* (Germar, 1839)

*Tetralonia fulvescens* Giraud, 1863

*Tetralonia graja* (Eversmann, 1852)

*Tetralonia inulae* Tkalců, 1979

*Tetralonia julliani* (Pérez, 1879)

*Tetralonia lyncea* Mocsáry, 1879

*Tetralonia malvae* (Rossi, 1790)

*Tetralonia mitsukurii* Cockerell, 1911

*Tetralonia nana* Morawitz, 1873

*Tetralonia pollinosa* (Lepeletier de Saint-Fargeau, 1841)

*Tetralonia salicariae* (Lepeletier de Saint-Fargeau, 1841)

*Tetralonia scabiosae* (Mocsáry, 1881)

*Tetralonia strigata* (Lepeletier de Saint-Fargeau, 1841)

*Tetralonia vicina* Morawitz, 1875

*Tetralonia yoshihiroi* (Ikudome, 2022)

**Tribe Anthophorini Dahlbom, 1835**

**Genus *Amegilla* Friese, 1897**

*Amegilla* (*Amegilla*) *garrula* (Rossi, 1790)

*Amegilla* (*Amegilla*) *ochroleuca* (Pérez, 1879)

*Amegilla* (*Amegilla*) *quadrifasciata* (de Villers, 1789)

*Amegilla* (*Glossamegilla*) *florea* (Smith, 1879)

*Amegilla* (*Micramegila*) *nigricornis* (Morawitz, 1872)

*Amegilla* (*Micramegilla*) *velocissima* (Fedtschenko, 1875)

*Amegilla* (*Zebramegilla*) *albigena* (Lepeletier de Saint-Fargeau, 1841)

*Amegilla* (*Zebramegilla*) *salviae* (Morawitz, 1875)

*Amegilla* (*Zebramegilla*) *savignyi* (Lepeletier, 1841)

**Genus *Anthophora* Latreille, 1803**

*Anthophora* (*Anthomegilla*) *arctica* Morawitz, 1883

*Anthophora* (*Anthophora*) *crinipes* Smith, 1854

*Anthophora* (*Anthophora*) *fulvitarsis* Brullé, 1832

*Anthophora* (*Anthophora*) *plumipes* (Pallas, 1772)

*Anthophora* (*Caranthophora*) *dufourii* Lepeletier de Saint-Fargeau, 1841

*Anthophora* (*Caranthophora*) *pubescens* (Fabricius, 1781)

*Anthophora* (*Clisodon*) *furcata* (Panzer, 1798)

*Anthophora* (*Clisodon*) *terminalis* Cresson, 1869

*Anthophora* (*Dasymegilla*) *quadrimaculata* (Panzer, 1798)

*Anthophora* (*Heliophila*) *bimaculata* (Panzer, 1798)

*Anthophora* (*Lophanthophora*) *affinis* Brullé, 1832

*Anthophora* (*Lophanthophora*) *atricilla* Eversmann, 1846

*Anthophora* (*Lophanthophora*) *robusta* (Klug, 1845)

*Anthophora* (*Lophanthophora*) *rutilans* Dours, 1870

*Anthophora* (*Melea*) *plagiata* (Illiger, 1806)

*Anthophora* (*Mystacanthophora*) *borealis* Morawitz, 1865

*Anthophora* (*Paramegilla*) *aeneiventris* Hedicke, 1931

*Anthophora* (*Paramegilla*) *astragali* Morawitz, 1878

*Anthophora* (*Paramegilla*) *balassogloi* (Radoszkowski, 1877)

*Anthophora* (*Paramegilla*) *deserticola* Morawitz, 1872

*Anthophora* (*Paramegilla*) *dubia* Eversmann, 1852

*Anthophora* (*Paramegilla*) *fulvipes* Eversmann, 1846

*Anthophora* (*Paramegilla*) *gracilipes* Morawitz, 1872

*Anthophora* (*Paramegilla*) *ireos* (Pallas, 1773)

*Anthophora* (*Paramegilla*) *podagra* Lepeletier de Saint-Fargeau, 1841

*Anthophora* (*Paramegilla*) *ponomarevae* Brooks, 1988

*Anthophora* (*Paramegilla*) *segnis* Eversmann, 1852

*Anthophora* (*Petalosternon*) *crassipes* Lepeletier de Saint-Fargeau, 1841

*Anthophora* (*Petalosternon*) *hanseni* Morawitz, 1883

*Anthophora* (*Petalosternon*) *radoszkowskyi* Fedtschenko, 1875

*Anthophora* (*Pyganthophora*) *aestivalis* (Panzer, 1801)

*Anthophora* (*Pyganthophora*) *altaica* Radoszkowski, 1882

*Anthophora* (*Pyganthophora*) *cincrea* (Friese, 1896)

*Anthophora* (*Pyganthophora*) *nigriceps* Morawitz, 1886

*Anthophora* (*Pyganthophora*) *orientalis* Morawitz, 1878

*Anthophora* (*Pyganthophora*) *pedata* Eversmann, 1852

*Anthophora* (*Pyganthophora*) *retusa* (Linnaeus, 1758)

*Anthophora* (*Pyganthophora*) *sichelii* Radoszkowski, 1869

*Anthophora* (*Pyganthophora*) *testaceipes* Morawitz, 1888

*Anthophora* (*Pyganthophora*) *vernalis* Morawitz, 1877

*Anthophora* (incertae sedis) *raddei* Morawitz, 1875

**Genus *Habropoda* Smith, 1854**

*Habropoda zonatula* Smith, 1854

**Tribe Melectini Westwood, 1839**

**Genus *Melecta* Latreille, 1802**

*Melecta* (*Melecta*) *albifrons* (Forster, 1771)

*Melecta* (*Melecta*) *amanda* Lieftinck, 1980

*Melecta* (*Melecta*) *baerii* (Radoszkowski, 1865)

*Melecta* (*Melecta*) *diacantha* Eversmann, 1852

*Melecta* (*Melecta*) *duodecimmaculata* (Rossi, 1790)

*Melecta* (*Melecta*) *eversmanni* Radoszkowski, 1893

*Melecta* (*Melecta*) *festiva* Lieftinck, 1980

*Melecta* (*Melecta*) *luctuosa* (Scopoli, 1770)

*Melecta* (*Melecta*) *mundula* Lieftinck, 1983

*Melecta* (*Melecta*) *sibirica* Radoszkowski, 1891

*Melecta* (*Melecta*) *tuberculata* Lieftinck, 1980

**Genus *Thyreomelecta* Rightmyer & Engel, 2003**

*Thyreomelecta propinqua* (Lieftinck, 1968)

*Thyreomelecta sibirica* (Radoszkowski, 1893)

**Genus *Thyreus* Panzer, 1806**

*Thyreus affinis* (Morawitz, 1873)

*Thyreus altaicus* (Radoszkowski, 1893)

*Thyreus decorus* (Smith, 1852)

*Thyreus hirtus* (de Beaumont, 1940)

*Thyreus histrionicus* (Illiger, 1806)

*Thyreus orbatus* (Lepeletier de Saint-Fargeau, 1841)

*Thyreus ramosus* (Lepeletier de Saint-Fargeau, 1841)

*Thyreus scutellaris* (Fabricius, 1781)

*Thyreus truncatus* (Pérez, 1883)

**Tribe Bombini Latreille, 1802**

**Genus *Bombus* Latreille, 1802**

*Bombus* (*Alpigenobombus*) *wurflenii* Radoszkowski, 1859

*Bombus* (*Alpinobombus*) *alpinus* (Linnaeus, 1758)

*Bombus* (*Alpinobombus*) *balteatus* Dahlbom, 1832

*Bombus* (*Alpinobombus*) *hyperboreus* Schönherr, 1809

*Bombus* (*Alpinobombus*) *pyrrhopygus* Friese, 1902

*Bombus* (*Bombias*) *confusus* Schenck, 1859

*Bombus* (*Bombus*) *cryptarum* (Fabricius, 1775)

*Bombus* (*Bombus*) *czerskianus* Vogt, 1911

*Bombus* (*Bombus*) *hypocrita* Pérez, 1905

*Bombus* (*Bombus*) *ignitus* Smith, 1869

*Bombus* (*Bombus*) *lucorum* (Linnaeus, 1761)

*Bombus* (*Bombus*) *magnus* Vogt, 1911

*Bombus* (*Bombus*) *patagiatus* Nylander, 1848

*Bombus* (*Bombus*) *sporadicus* Nylander, 1848

*Bombus* (*Bombus*) *terrestris* (Linnaeus, 1758)

*Bombus* (*Cullumanobombus*) *cullumanus* (Kirby, 1802)

*Bombus* (*Cullumanobombus*) *semenoviellus* Skorikov, 1910

*Bombus* (*Cullumanobombus*) *unicus* Morawitz, 1883

*Bombus* (*Kallobombus*) *soroeensis* (Fabricius, 1777)

*Bombus* (*Megabombus*) *argillaceus* (Scopoli, 1763)

*Bombus* (*Megabombus*) *consobrinus* Dahlbom, 1832

*Bombus* (*Megabombus*) *czerskii* Skorikov, 1910

*Bombus* (*Megabombus*) *diversus* Smith, 1869

*Bombus* (*Megabombus*) *gerstaeckeri* Morawitz, 1881

*Bombus* (*Megabombus*) *hortorum* (Linnaeus, 1761)

*Bombus* (*Megabombus*) *portchinsky* Radoszkowski, 1883

*Bombus* (*Megabombus*) *ruderatus* (Scopoli, 1763)

*Bombus* (*Megabombus*) *saltuarius* (Skorikov, 1931)

*Bombus* (*Megabombus*) *tichenkoi* (Skorikov, 1926)

*Bombus* (*Megabombus*) *ussurensis* Radoszkowski, 1877

*Bombus* (*Melanobombus*) *alagesianus* Reinig, 1930

*Bombus* (*Melanobombus*) *eriophorus* Klug, 1807

*Bombus* (*Melanobombus*) *incertoides* Vogt, 1911

*Bombus* (*Melanobombus*) *lapidarius* (Linnaeus, 1758)

*Bombus* (*Melanobombus*) *sichelii* Radoszkowski, 1860

*Bombus* (*Mendacibombus*) *margreiteri* Skorikov, 1910

*Bombus* (*Mendacibombus*) *handlirschianus* Vogt, 1909

*Bombus* (*Psithyrus*) *barbutellus* (Kirby, 1802)

*Bombus* (*Psithyrus*) *bohemicus* Seidl, 1837

*Bombus* (*Psithyrus*) *branickii* (Radoszkowski, 1893)

*Bombus* (*Psithyrus*) *campestris* (Panzer, 1801)

*Bombus* (*Psithyrus*) *flavidus* Eversmann, 1852

*Bombus* (*Psithyrus*) *norvegicus* (Sparre-Schneider, 1918)

*Bombus* (*Psithyrus*) *quadricolor* (Lepeletier de Saint-Fargeau, 1832)

*Bombus* (*Psithyrus*) *rupestris* (Fabricius, 1793)

*Bombus* (*Psithyrus*) *sylvestris* (Lepeletier de Saint-Fargeau, 1832)

*Bombus* (*Psithyrus*) *vestalis* (Fourcroy, 1785)

*Bombus* (*Pyrobombus*) *ardens* Smith, 1879

*Bombus* (*Pyrobombus*) *beaticola* (Tkalců, 1968)

*Bombus* (*Pyrobombus*) *biroi* Vogt, 1911

*Bombus* (*Pyrobombus*) *brodmannicus* Vogt, 1909

*Bombus* (*Pyrobombus*) *cingulatus* Wahlberg, 1854

*Bombus* (*Pyrobombus*) *glacialis* Friese, 1902

*Bombus* (*Pyrobombus*) *haematurus* Kriechbaumer, 1870

*Bombus* (*Pyrobombus*) *hypnorum* (Linnaeus, 1758)

*Bombus* (*Pyrobombus*) *jonellus* (Kirby, 1802)

*Bombus* (*Pyrobombus*) *koropokkrus* Sakagami & Ishikawa, 1972

*Bombus* (*Pyrobombus*) *lapponicus* (Fabricius, 1793)

*Bombus* (*Pyrobombus*) *modestus* Eversmann, 1852

*Bombus* (*Pyrobombus*) *monticola* Smith, 1849

*Bombus* (*Pyrobombus*) *oceanicus* Friese, 1909

*Bombus* (*Pyrobombus*) *pratorum* (Linnaeus, 1761)

*Bombus* (*Sibiricobombus*) *asiaticus* Morawitz, 1875

*Bombus* (*Sibiricobombus*) *niveatus* Kriechbaumer, 1870

*Bombus* (*Sibiricobombus*) *sibiricus* (Fabricius, 1781)

*Bombus* (*Subterraneobombus*) *amurensis* Radozkowski, 1862

*Bombus* (*Subterraneobombus*) *distinguendus* Morawitz, 1868

*Bombus* (*Subterraneobombus*) *fragrans* (Pallas, 1771)

*Bombus* (*Subterraneobombus*) *melanurus* Lepeletier de Saint-Fargeau, 1836

*Bombus* (*Subterraneobombus*) *subterraneus* (Linnaeus, 1758)

*Bombus* (*Subterraneobombus*) *tshitscherini* Radoszkowski, 1862

*Bombus* (*Thoracobombus*) *anachoreta* (Skorikov, 1914)

*Bombus* (*Thoracobombus*) *armeniacus* Radozkowski, 1877

*Bombus* (*Thoracobombus*) *deuteronymus* Schulz, 1906

*Bombus* (*Thoracobombus*) *exil* Skorikov, 1922

*Bombus* (*Thoracobombus*) *filchnerae* Vogt, 1908

*Bombus* (*Thoracobombus*) *humilis* Illiger, 1806

*Bombus* (*Thoracobombus*) *laesus* Morawitz, 1875

*Bombus* (*Thoracobombus*) *mesomelas* Gerstaecker, 1869

*Bombus* (*Thoracobombus*) *mlokosievitzii* Radoszkowski, 1877

*Bombus* (*Thoracobombus*) *muscorum* (Linnaeus, 1758)

*Bombus* (*Thoracobombus*) *pascuorum* (Scopoli, 1763)

*Bombus* (*Thoracobombus*) *persicus* Radoszkowski, 1881

*Bombus* (*Thoracobombus*) *pomorum* (Panzer, 1805)

*Bombus* (*Thoracobombus*) *pseudobaicalensis* Vogt, 1911

*Bombus* (*Thoracobombus*) *ruderarius* (Müller, 1776)

*Bombus* (*Thoracobombus*) *schrencki* Morawitz, 1881

*Bombus* (*Thoracobombus*) *sylvarum* (Linnaeus, 1761)

*Bombus* (*Thoracobombus*) *tricornis* Radoszkowski, 1888

*Bombus* (*Thoracobombus*) *velox* (Skorikov, 1914)

*Bombus* (*Thoracobombus*) *veteranus* (Fabricius, 1793)

*Bombus* (*Thoracobombus*) *zonatus* Smith, 1854

**Tribe Apini Latreille, 1802**

**Genus *Apis* Linnaeus, 1758**

*Apis cerana* Fabricius, 1893

*Apis mellifera* Linnaeus, 1758
